# *Bacteroides Fragilis‐*Derived Outer Membrane Vesicles Deliver MiR‐5119 and Alleviate Colitis by Targeting PD‐L1 to Inhibit GSDMD‐Mediated Neutrophil Extracellular Trap Formation

**DOI:** 10.1002/advs.202500781

**Published:** 2025-06-25

**Authors:** Yi Yang, Lanmengxi Yang, Yilin Yang, Haiyi Deng, Shiyu Su, Yinxiao Xia, Jin Su, Yuheng Liu, Junwei Wu, Jing Zhang, Yao Liao, Lifu Wang

**Affiliations:** ^1^ KingMed School of Laboratory Medicine The Affiliated Traditional Chinese Medicine Hospital Guangzhou Medical University Guangzhou 511436 China; ^2^ Engineering Technology Research Center of Intelligent Diagnosis for Infectious Diseases in Guangdong Province Guangzhou 511436 China; ^3^ Guangzhou Key Laboratory for Clinical Rapid Diagnosis and Early Warning of Infectious Diseases Guangzhou 511436 China; ^4^ The First Affiliated Hospital of Guangzhou Medical University Guangzhou 510120 China

**Keywords:** bacteroides fragilis, colitis, inflammatory bowel disease, miR‐5119, neutrophil extracellular traps, outer membrane vesicles

## Abstract

Inflammatory bowel disease (IBD) results from a breakdown in the symbiotic relationship between the intestinal commensal microflora and the mucosal immune system. Non‐toxigenic *Bacteroides fragilis*, a common human colon symbiote, has been shown to alleviate colitis. However, the underlying mechanisms of this alleviation remain incompletely understood. Herein, it is demonstrated that promoting the secretion of *B. fragilis* outer membrane vesicles (Bf^OMVs+^) enhances its ability to alleviate dextran sodium sulfate (DSS)‐induced colitis, while inhibiting *B. fragilis* OMV secretion (Bf^OMVs−^) reduces this effect. Bf^OMVs+^ alleviates colitis by inhibiting neutrophil recruitment and neutrophil extracellular trap (NET) formation. Further, *B. fragilis* OMVs (Bf‐OMVs) are isolated and extracted, then administered them intraperitoneally to DSS‐induced colitis mice, observing that Bf‐OMVs can target intestinal tissues, the spleen, and bone marrow, and they are internalized by neutrophils to inhibit NET formation, thereby alleviating colitis. The expression profile of miRNAs in Bf‐OMVs is assessed, revealing that Bf‐OMVs are enriched with mmu‐miR‐like sRNA, miR‐5119, which targets and inhibits PD‐L1, leading to the suppression of GSDMD‐mediated NET release and promoting the proliferation of intestinal stem cells (ISCs), culminating in the alleviation of colitis. These findings provide new insights into the role of *B. fragilis* OMVs in the pathogenesis and treatment of IBD.

## Graphical abstract

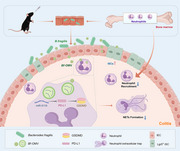

## 1 Introduction

Inflammatory bowel disease (IBD), which encompasses ulcerative colitis (UC) and Crohn's disease (CD), results from a breakdown in the symbiotic relationship between the intestinal commensal microflora and the mucosal immune system.^[^ ^1^ ^]^ Both adaptive and innate immunity are involved in this chronic inflammatory process.^[^ ^2^ ^]^ Studies have demonstrated that signals from microbial metabolites influence immune maturation, immune homeostasis, host energy metabolism, and the maintenance of mucosal integrity. In numerous investigations of IBD, alterations in the composition and function of the microbiome have been described.^[^ ^3^ ^]^

Dysbiosis (imbalances in the microbiota) is hypothesized to be a major factor in human disorders such as IBD. *Bacteroides fragilis*, a common resident of the human gut, has been extensively studied in this context.^[^ ^4^ ^]^ Studies have demonstrated that different strains of *B. fragilis* can play distinct roles in human health and disease. Enterotoxigenic *B. fragilis* (ETBF) is known to promote intestinal inflammation and malignancy,^[^ ^5^ ^]^ while non‐toxigenic *B. fragilis* (NTBF), a human colon symbiote, has been proposed as a probiotic. Although ETBF stimulates chronic inflammation and participates in colorectal carcinogenesis, the majority of *B. fragilis* strains are non‐toxigenic and provide beneficial effects to the host, including the alleviation of colitis.^[^ ^6^ ^]^ Studies have demonstrated that *B. fragilis* alleviates colitis by modulating host immune responses.^[^ ^7^, ^8^, ^9^ ^]^ In CD45RB^hi^ T‐cell transfer‐or 2,4,6‐trinitrobenzene sulfonic acid (TNBS)‐induced colitis models, *B. fragilis* has been shown to protect against colitis by inducing interleukin‐10 production.^[^ ^7^, ^10^ ^]^

Gram‐negative bacteria typically release outer membrane vesicles (OMVs) during growth, and these vesicles play a crucial role in microbe‐microbe and host‐microbe interactions.^[^ ^11^ ^]^ Recent studies conducted in mice have highlighted the roles of OMVs produced by bacteria in eliciting immunostimulatory responses by bypassing host barriers.^[^ ^12^, ^13^ ^]^ OMVs can contain adhesins, sulfatases, and proteases that facilitate their interaction with host cells, allowing them to enter these cells through various pathways, including microvesicle endocytosis, lipid raft‐dependent endocytosis, and clathrin‐dependent endocytosis.^[^ ^14^ ^]^

Among the various immune cells involved in IBD, neutrophils are the first to infiltrate and sustain inflammation by releasing pro‐inflammatory cytokines and chemokines.^[^ ^15^ ^]^ Neutrophils are key immune cells contributing to dysregulated mucosal immune responses in IBD, associated with architectural distortion of tissue, crypt destruction, and crypt abscess formation.^[^ ^16^ ^]^ In addition to basic effector mechanisms, such as phagocytosis and chemotaxis, neutrophils can form extracellular traps (NETs), which are composed of a reticular structure containing chromatin (DNA and histones), granule proteins, and enzymes such as myeloperoxidase (MPO) and neutrophil elastase (NE). These NETs act as traps that induce extracellular pathogen death and/or may exacerbate tissue damage.^[^ ^15^ ^]^ Recent evidence indicates that NETs play an important and significant role in the pathogenesis of IBD. In the dextran sodium sulfate (DSS)‐induced colitis model, the intestinal barrier is damaged by NETs via the induction of enterocyte apoptosis.^[^ ^17^ ^]^ Increased NET‐associated protein expression has been confirmed in the inflammatory tissues of patients with UC.^[^ ^18^ ^]^ Therefore, downregulating NET formation can help alleviate colitis.^[^ ^19^ ^]^

In this study, we demonstrated that *B. fragilis* alleviates DSS‐induced colitis through the secretion of OMVs (Bf‐OMVs). Mechanistically, Bf‐OMVs are enriched with miR‐5119, which targets and inhibits PD‐L1, leading to the suppression of GSDMD‐mediated NET release, thereby alleviating colitis.

## 2 Results

### 2.1 OMVs are Involved in the Remission of DSS‐Induced Colitis by B. Fragilis

To investigate whether OMVs are involved in the alleviation of IBD by *B. fragilis*, we used monensin to stimulate *B. fragilis* and promote its OMV secretion while using GW4869 to inhibit OMV secretion from *B. fragilis*.^[^ ^20^, ^21^ ^]^ Subsequently, we examined whether monensin and GW4869 affect bacterial viability or modulate OMV secretion (Figure S1a, Supporting Information). As shown in Figure S1b (Supporting Information), no statistically significant differences in bacterial colony counts were observed among vehicle‐, monensin‐, or GW4869‐treated *B. fragilis* after 24 h of culture on agar plates, indicating that neither monensin nor GW4869 impairs bacterial viability. Figure S1c (Supporting Information) demonstrates that monensin significantly enhanced OMV production, whereas GW4869 markedly suppressed OMV secretion, as evidenced by reduced total protein content in isolated OMVs. Consistent with these protein‐level changes, nanoflow cytometry analyses revealed a substantial increase in OMV particle counts following monensin treatment and a pronounced decrease after GW4869 administration (Figure S1d,e, Supporting Information).

**Figure 1:**
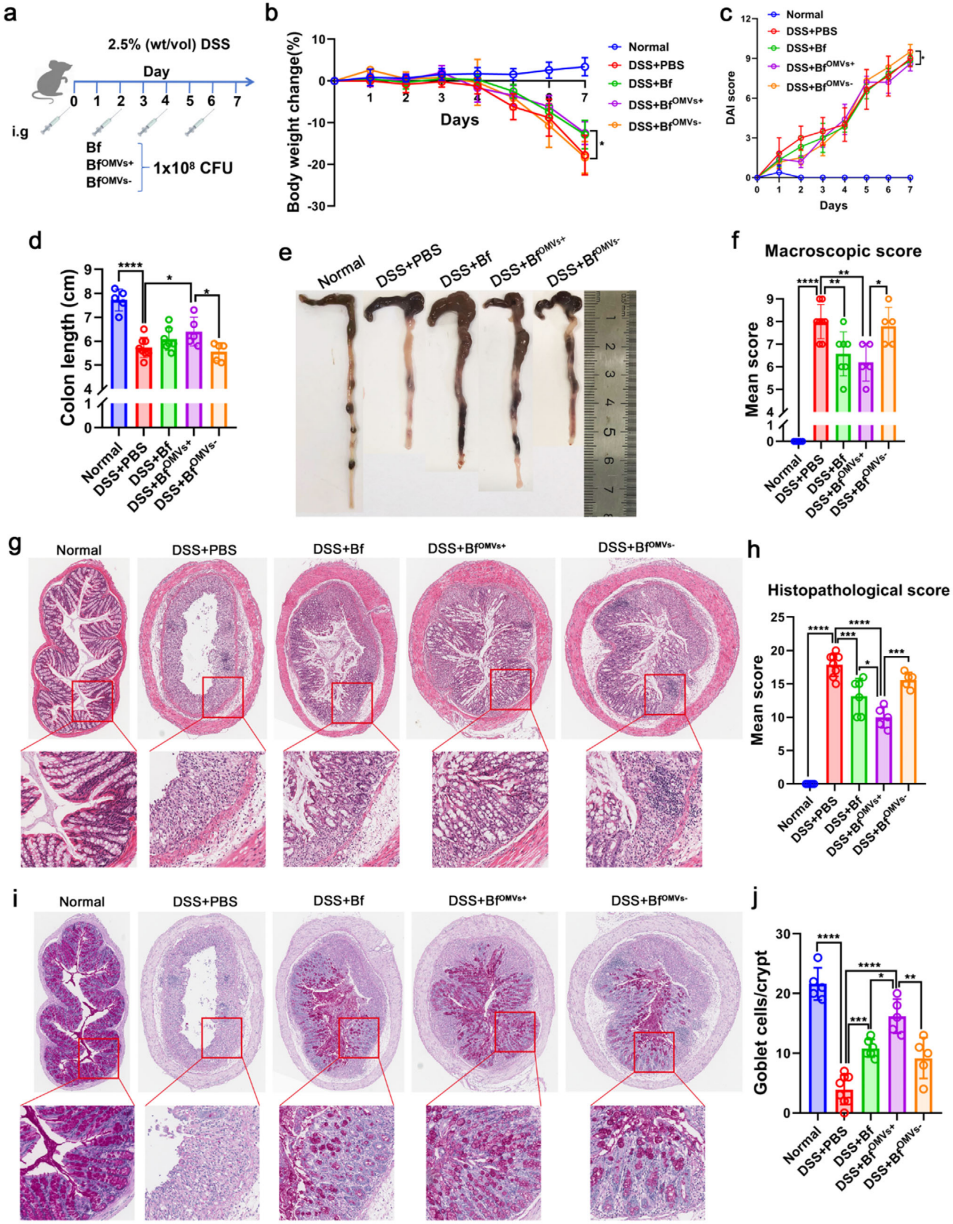
Promoting *B. fragilis* OMV secretion enhances its ability to alleviate colitis while inhibiting OMV secretion reduces this effect. a) Mice with DSS‐induced colitis were treated with *B. fragilis*, the OMV‐producing strain (Bf^OMVs+^), and the OMV‐inhibited strain (Bf^OMVs−^). b) Body weight changes in the experimental groups, normalized to Day 0 body weight. c) Disease activity index (DAI) scores are based on diarrhea, bleeding, and body weight loss. d) Measurement of colon length from mice. e) Macroscopic appearance of the colon, as represented by the colon with the mean colon length and typical injury findings. f) Macroscopic colon scores indicate the colon injury's severity. g) Histopathological examination of colon tissues stained with H&E. h) Histopathological scores for the colon tissue samples were determined by H&E staining. i, j) Alcian blue‐periodic acid‐Schiff (AB‐PAS) staining used to assess goblet cell depletion (i), with goblet cell counts presented (j). n = 5–7; results are presented as the mean ± SD; ^*^ *p* < 0.05, ^**^ *p* < 0.01, ^***^ *p* < 0.001, ^****^ *p* < 0.0001.

Next, we treated DSS‐induced colitis in mice with *B. fragilis*, the OMV‐producing strain (Bf^OMVs+^), and the OMV‐inhibited strain (Bf^OMVs−^) and evaluated their therapeutic effects (**Figure** **1**a). Compared with the Bf^OMVs−^‐treated group, the Bf^OMVs+^‐treated group exhibited a more significant weight loss alleviation in mice with colitis (Figure 1b). Similarly, the disease activity index (DAI) was markedly reduced in the Bf^OMVs+^‐treated group compared with the Bf^OMVs−^‐treated group (Figure 1c). Additionally, Bf^OMVs+^ significantly increased the colonic length in mice with colitis, while the colonic length was not notably increased in the *B. fragilis*‐treated or Bf^OMVs−^‐treated groups (Figure 1d,e). Furthermore, both *B. fragilis* and Bf^OMVs+^ significantly decreased the macroscopic colon scores in mice, with Bf^OMVs+^ showing a more pronounced reduction compared with the Bf^OMVs−^‐treated group, which did not differ significantly from the PBS‐treated group (Figure 1f). Histological analyses revealed that Bf^OMVs+^ treatment significantly alleviated the substantial disruption of colonic architecture caused by DSS, which is characterized by neutrophil and lymphohistiocyte infiltration, crypt loss, crypt abscess formation, submucosal edema, and goblet cell depletion, with a more significant effect compared with *B. fragilis* and Bf^OMVs−^treatment (Figure 1g). Consistently, histological scores were significantly reduced in Bf^OMVs+^‐treated mice, with a more pronounced reduction compared with *B. fragilis* and Bf^OMVs−^treatment (Figure 1h). Similarly, goblet cell depletion was significantly alleviated following Bf^OMVs+^ treatment, with a more significant effect compared with *B. fragilis* and Bf^OMVs−^treatment (Figure 1i,j). These results indicate that OMVs are involved in the remission of DSS‐induced colitis by *B. fragilis*.

### 2.2 Bf^OMVs+^ Inhibits Neutrophil Infiltration and NET Formation in Mice with Colitis

**Figure 2:**
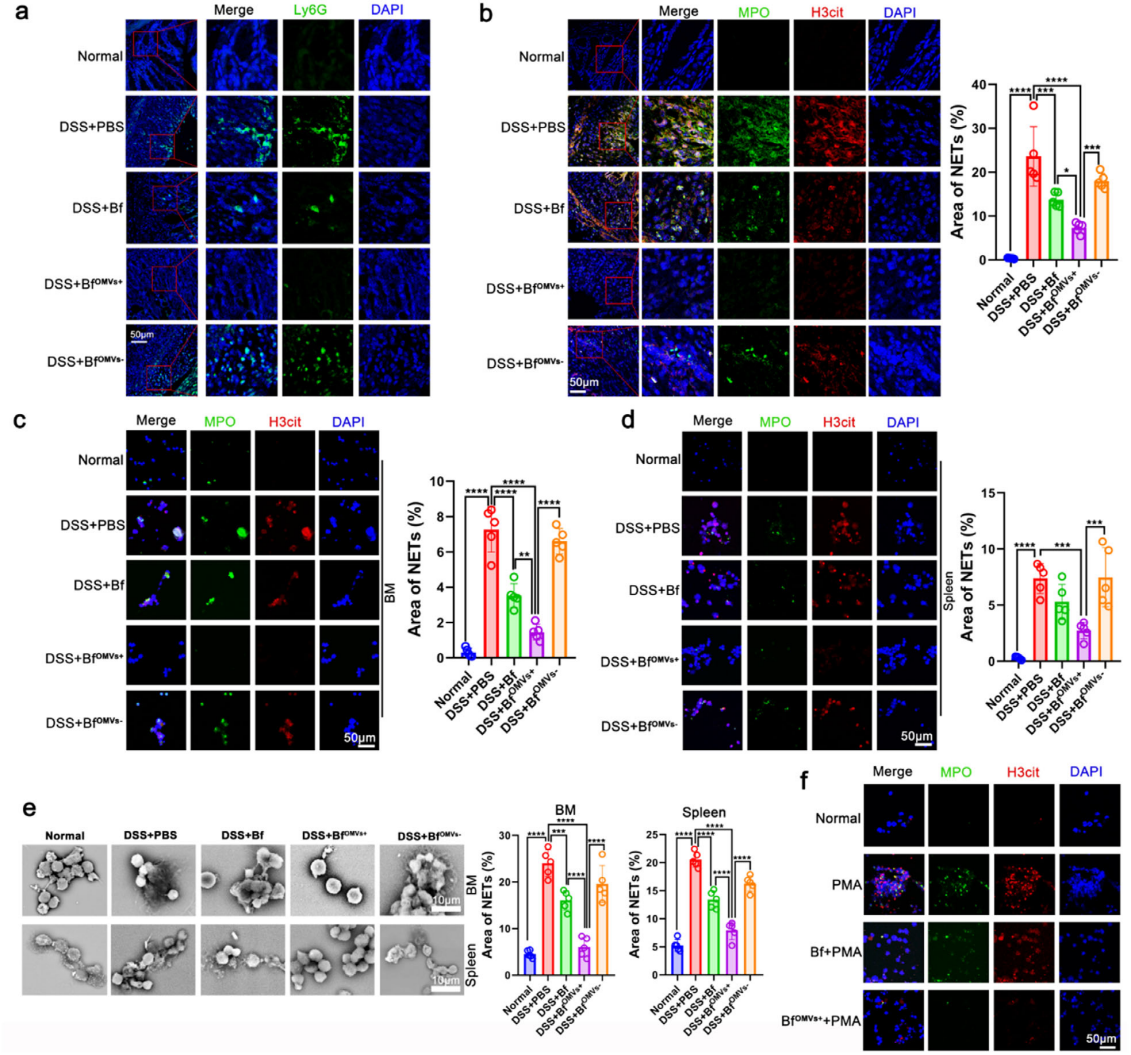
Bf^OMVs+^ inhibits neutrophil infiltration and NET formation in mice with colitis. a) Immunofluorescence analyses of neutrophil levels in the colon tissue of mice. b) Immunofluorescence analysis was used to evaluate neutrophil extracellular traps (NETs) formation in colon tissues. Co‐localization of H3cit and MPO was used as markers for NETs detection. c–e) Neutrophils isolated from BM and spleens of mice were cultured in vitro for 24 h. Immunofluorescence analyses and scanning electron microscopy (SEM) were used to assess NET formation. f) Neutrophils from the bone marrow (BM) of normal mice were treated with *B. fragilis* and Bf^OMVs+^ while using PMA to induce NET formation in vitro. NETs were assessed by immunofluorescence and SD. n = 5; results are presented as the mean ± SD; ^*^ *p* < 0.05, ^**^ *p* < 0.01, ^***^ *p* < 0.001, ^****^ *p* < 0.0001.

Neutrophil infiltration of the intestinal mucosa is a hallmark of active IBD. In DSS‐induced colitis mice, significant neutrophil infiltration into the intestinal mucosa was observed, which was significantly decreased after treatment with *B. fragilis* (**Figure** **2**a). Interestingly, compared with *B. fragilis* treatment, Bf^OMVs+^ treatment further downregulated neutrophil infiltration in mice with colitis, while the reduction in neutrophil infiltration was reduced after Bf^OMVs−^treatment (Figure 2a). Bone marrow (BM) neutrophil levels were markedly lower in DSS‐induced colitis mice compared with normal mice (Figures S2 and S3a,b). This depletion was likely due to the recruitment of neutrophils to peripheral organs during acute colitis. We observed that *B. fragilis* and Bf^OMVs+^ treatment alleviated this peripheral neutrophil recruitment, consequently restoring neutrophil levels in the BM, whereas Bf^OMVs−^treatment did not possess this ability (Figure S3a,b, Supporting Information). NETs can promote IBD progression.^[^ ^15^ ^]^ Immunofluorescence analyses revealed a significant increase in NET formation in the intestinal mucosa of mice with colitis, which was significantly decreased following Bf^OMVs+^ treatment, with a more pronounced reduction compared with *B. fragilis* and Bf^OMVs−^treatment (Figure 2b). To determine whether this downregulation of NET formation was due solely to reduced neutrophil counts or also to the direct inhibition of NET formation by neutrophils, we cultured neutrophils isolated from the BM and spleens of mice in vitro. We found that neutrophils from mice with colitis easily form NETs in vitro, while NET formation was significantly downregulated in neutrophils from Bf^OMVs+^‐treated mice, with a lower level of NET formation compared with *B. fragilis* and Bf^OMVs−^treatment (Figure 2c–e). Phorbol myristate acetate (PMA) is an inducer of NET formation. Comparatively, neutrophils from PBS‐treated mice with colitis were more susceptible to PMA‐induced NET formation in vitro than those from *B. fragilis‐*, Bf^OMVs+^‐, and Bf^OMVs−^‐treated mice with colitis, with a less pronounced PMA‐induced NET formation in the Bf^OMVs+^ treatment group (Figure S4a–d, Supporting Information). Additionally, we isolated BM neutrophils from normal mice and treated them with *B. fragilis* and Bf^OMVs+^ while using PMA to induce NET formation in vitro. We found that *B. fragilis* and Bf^OMVs+^ significantly inhibited PMA‐induced NET formation, inhibition with a more pronounced effect by Bf^OMVs+^ (Figure 2f; Figure S4e, Supporting Information). These results suggest that Bf^OMVs+^ significantly inhibit neutrophil infiltration and NET formation.

### 2.3 Bf‐OMVs are Internalized by Neutrophils and Inhibit NET Formation In Vitro

**Figure 3:**
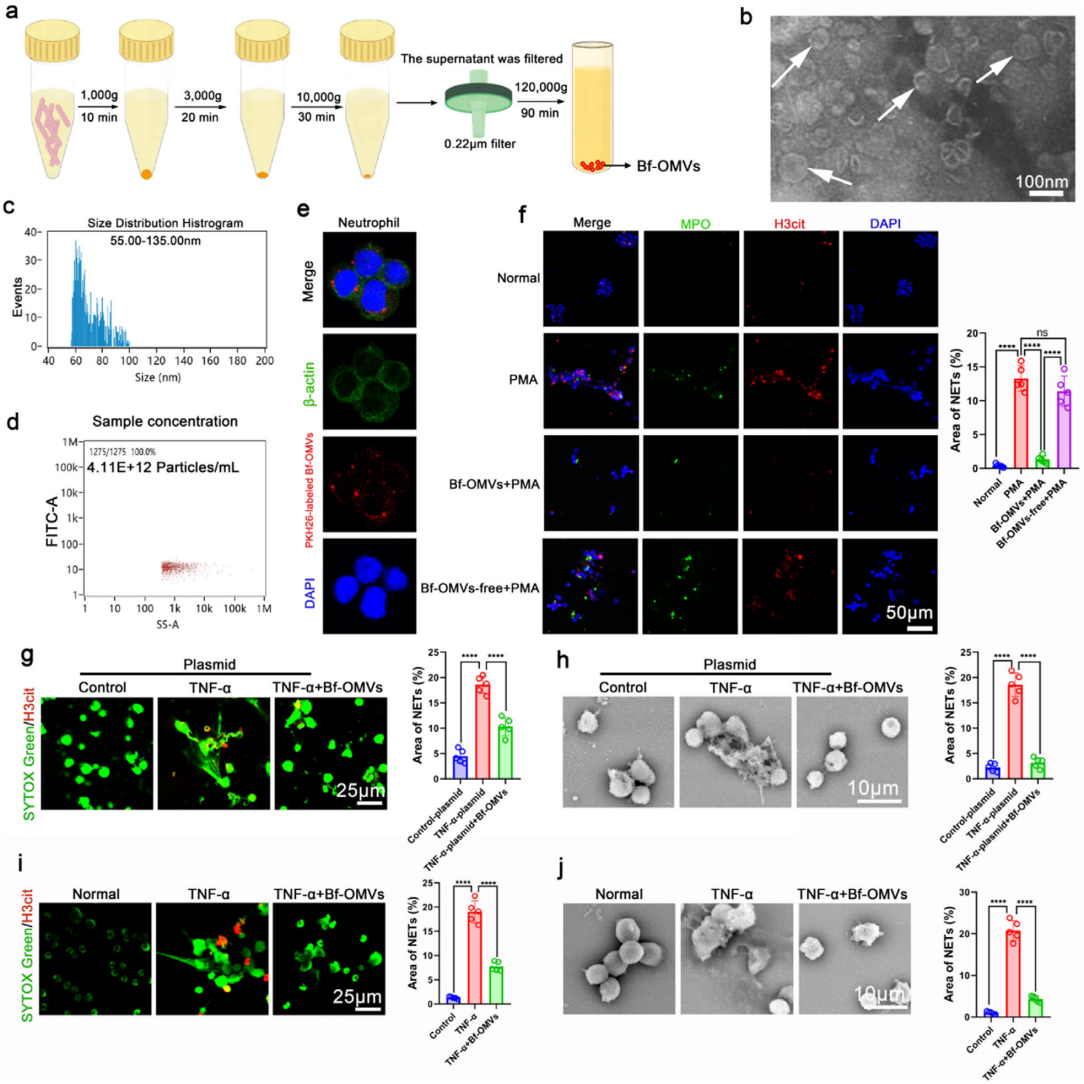
Bf‐OMVs are internalized by neutrophils and inhibit NET formation in vitro. a) Bf‐OMVs were isolated from the culture supernatant of *B. fragilis* using a multi‐step differential centrifugation process. b) Transmission electron microscopy (TEM) revealed the cup‐shaped morphology of Bf‐OMVs. c, d) Nanoflow cytometry analysis was used to determine the size distribution (c) and concentration (d) of Bf‐OMVs. e) Neutrophils were incubated with PKH26‐labeled Bf‐OMVs, and Bf‐OMV internalization was visualized by laser scanning confocal microscopy. f) Neutrophils from the BMs of normal mice were treated with Bf‐OMVs while using PMA to induce NET formation in vitro. NETs were assessed by immunofluorescence. g, h) BM neutrophils transfected with TNF‐α overexpression or control plasmids and treated with Bf‐OMVs; NETs were analyzed by SYTOX Green/H3cit co‐staining and SEM. i, j) BM neutrophils treated with Bf‐OMVs and TNF‐α; NETs were assessed by immunofluorescence and SEM. n = 5; results are presented as the mean ± SD; ^*^ *p* < 0.05, ^**^ *p* < 0.01, ^***^ *p* < 0.001, ^****^ *p* < 0.0001; ns: non‐significance.

Next, we investigated whether *B. fragilis* exerted its effects by secreting OMVs that directly act on neutrophils to inhibit NET formation. Bf‐OMVs were isolated from the culture supernatant of *B. fragilis* using a multi‐step differential centrifugation protocol (**Figure** **3**a). Transmission electron microscopy (TEM) revealed that the isolated Bf‐OMVs exhibited the characteristic cup‐shaped morphology (Figure 3b). Nanoflow cytometry analyses revealed that the size distribution of the isolated Bf‐OMVs ranged from 55 to 135 nm in diameter (Figure 3c), with a concentration of ≈ 4.11 × 10^12^ particles mL^−1^ (Figure 3d). Subsequently, we isolated BM neutrophils from normal mice and treated them with Bf‐OMVs while using PMA to induce NET formation in vitro. We found that Bf‐OMVs were internalized by neutrophils (Figure 3e) and significantly inhibited PMA‐induced NET formation (Figure 3f). TNF‐α can induce NET formation.^[^ ^22^ ^]^ We constructed TNF‐α overexpression plasmids and transfected them into neutrophils (Figure S5, Supporting Information). SYTOX Green was used to detect extracellular trap formation.^[^ ^23^ ^]^ Using immunostaining (SYTOX Green and H3cit co‐staining) and SEM, we observed that Bf‐OMVs significantly inhibited TNF‐α‐induced NET formation (Figure 3g,h). Furthermore, exogenous TNF‐α stimulation experiments corroborated that Bf‐OMVs significantly inhibited TNF‐α‐induced NET generation (Figure 3i,j). These results suggest that Bf‐OMVs are internalized by neutrophils and inhibit NET formation.

### 2.4 Bf‐OMVs Relieved DSS‐Induced Colitis

**Figure 4:**
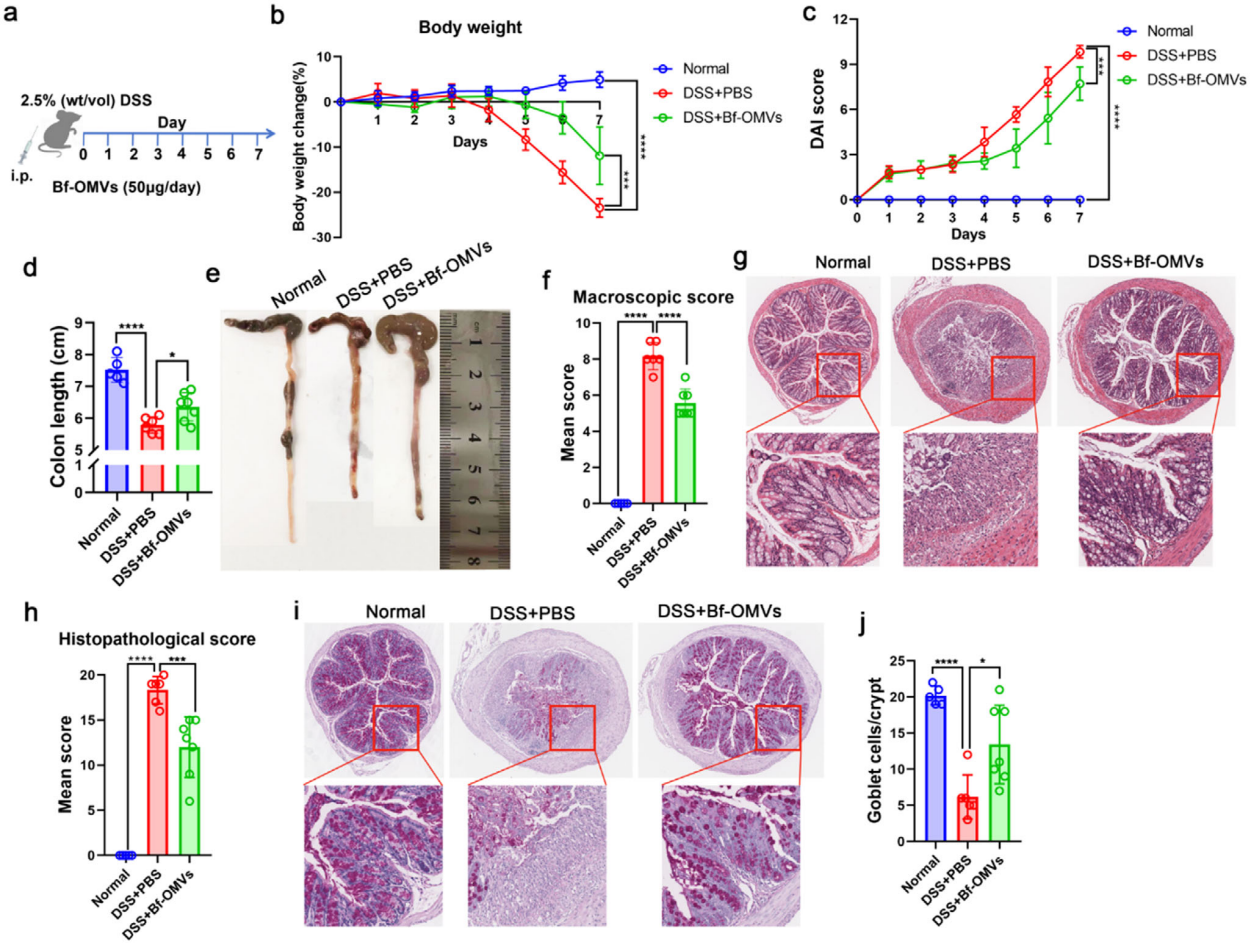
Bf‐OMVs relieved DSS‐induced colitis. a) Mice with DSS‐induced colitis were treated with intraperitoneal injections of Bf‐OMVs. b) Body weight changes in the experimental groups, normalized to day 0 body weight. c) DAI scores. d) Colon length measurements from mice in different treatment groups. e) Macroscopic appearance of the colon. f) Macroscopic colon scores indicate the severity of the colon injury. g) Histopathological examination of colon tissues stained with H&E. h) Histopathological scores for the colon tissue samples were determined by H&E staining. i, j) Alcian blue‐periodic acid‐Schiff (AB‐PAS) staining used to assess goblet cell depletion, with goblet cell counts presented. n = 5–7; results are presented as the mean ± SD; ^*^ *p* < 0.05, ^***^ *p* < 0.001, ^****^ *p* < 0.0001.

We investigated whether Bf‐OMVs can alleviate the symptoms of DSS‐induced colitis in mice. Mice with DSS‐induced colitis were treated with intraperitoneal injections of Bf‐OMVs (**Figure** **4**a). As shown in Figure 4b, compared with the DSS+PBS group, the DSS+Bf‐OMVs group exhibited significantly reduced body weight loss beginning on day 5. The DAI of the DSS+Bf‐OMVs group was consistently lower than that of the DSS+PBS group from day 4 onward (Figure 4c). The DSS+Bf‐OMVs group displayed significantly greater colon lengths compared with the DSS+PBS group (Figure 4d,e). Similarly, macroscopic colon scores in the DSS+Bf‐OMVs group were significantly lower than those in the DSS+PBS group (Figure 4f). Histological analyses revealed that Bf‐OMV treatment markedly mitigated colonic architecture disruptions compared with the DSS+PBS group (Figure 4g). Consistently, histological scores were significantly reduced in Bf‐OMV‐treated mice (Figure 4h), and goblet cell depletion was significantly alleviated following Bf‐OMV treatment (Figure 4i,j). These results suggest that Bf‐OMVs protect against DSS‐induced colitis in mice.

Additionally, mice with DSS‐induced colitis were treated with Bf‐OMVs via oral gavage (Figure S6a, Supporting Information). Comprehensive assessments of body weight loss, DAIs, colon lengths, macroscopic colon scores, histological analyses, histological scores, and goblet cell depletion demonstrated that Bf‐OMVs administered through oral gavage also protected against DSS‐induced colitis in mice (Figure S6b–j, Supporting Information).

### 2.5 Bf‐OMVs Inhibit Neutrophil Recruitment and NET Formation to Alleviate DSS‐Induced Colitis

**Figure 5:**
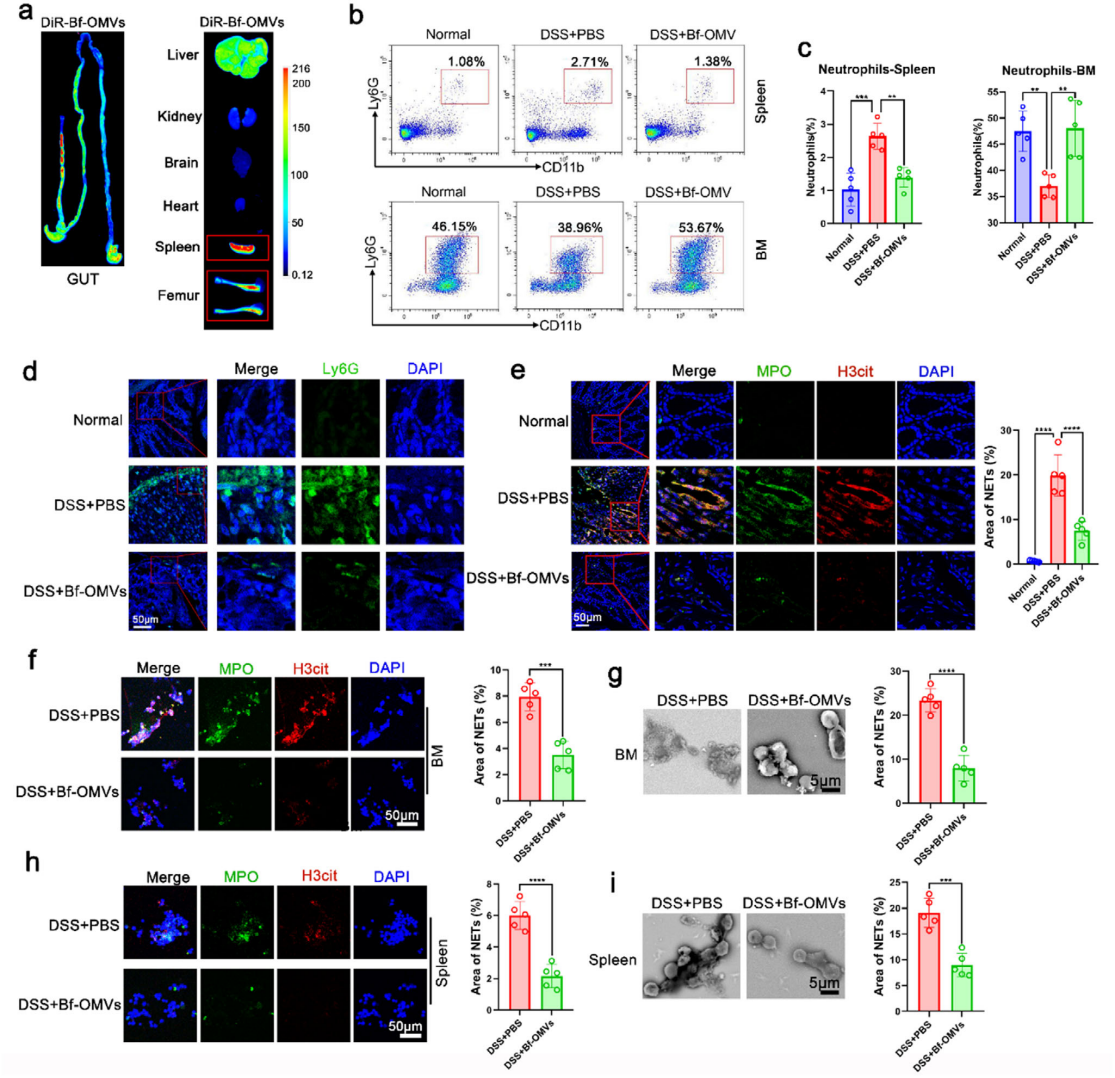
Bf‐OMVs inhibit neutrophil recruitment and NET formation to alleviate DSS‐induced colitis. a) DiR‐labeled Bf‐OMVs were intraperitoneally injected into mice, and the biodistribution of Bf‐OMVs was assessed in the intestinal tissues, liver, kidneys, brain, heart, spleen, and femur after 24 h post‐injection. b, c) The percentages of neutrophils in the spleens and BMs were analyzed by flow cytometric analyses (b), and the results of the statistical analyses are shown (c). d) Immunofluorescence analyses of neutrophil levels in the colon tissue of mice. e) Immunofluorescence analyses were used to evaluate NET formation in colon tissues. f–i) Neutrophils isolated from the BMs and spleens of mice were cultured in vitro for 24 h. Immunofluorescence analyses (f, h) and SEM (g, i) were used to assess NET formation. n = 5–7; results are presented as the mean ± SD; ^*^ *p* < 0.05, ^**^ *p* < 0.01, ^***^ *p* < 0.001, ^****^ *p* < 0.0001.

To explore whether the therapeutic effect of Bf‐OMVs on colitis is related to neutrophil regulation, we intraperitoneally injected DiR‐labeled Bf‐OMVs into mice and observed the distribution of Bf‐OMVs in the tissues 24 h later. Bf‐OMVs were predominantly found in the intestine, spleen, and femur (**Figure** **5**a). Therefore, we evaluated neutrophil levels in the spleen, BM, and intestine. We observed that neutrophil levels in the spleen and colon of Bf‐OMVs‐treated mice with colitis were significantly lower than those in PBS‐treated mice (Figure 5b–d). Additionally, Bf‐OMV treatment alleviated peripheral neutrophil recruitment, leading to the restoration of neutrophil levels in the BM (Figure 5b). We further examined NET formation in the intestinal mucosa of mice with colitis. Immunofluorescence analyses revealed that NET formation in the intestinal mucosa was significantly decreased following Bf‐OMV treatment (Figure 5e). To determine if this downregulation of NET formation was due solely to reduced neutrophil numbers or an inhibitory effect of neutrophils, we cultured neutrophils isolated from the BMs and spleens of mice in vitro. We found that NET formation was significantly downregulated in neutrophils from Bf‐OMVs‐treated mice (Figure 5f‐i). Similarly, neutrophils from PBS‐treated mice with colitis were more susceptible to PMA‐induced NET formation in vitro compared with those from Bf‐OMVs‐treated mice with colitis (Figure S7a–c, Supporting Information). These results suggest that Bf‐OMVs inhibit neutrophil recruitment and NET formation in mice with colitis.

Additionally, we observed that neutrophil levels in the colon of Bf‐OMV‐treated mice administered via oral gavage were significantly reduced compared to PBS‐treated mice (Figure S8a,b, Supporting Information). Bf‐OMV treatment through oral gavage also alleviated peripheral neutrophil recruitment, restoring neutrophil levels in the BM (Figure S8a,b, Supporting Information). Moreover, NET formation was markedly downregulated in neutrophils from Bf‐OMV‐treated mice administered via oral gavage (Figure S8c,d, Supporting Information). These results indicate that Bf‐OMVs administered through oral gavage inhibit neutrophil recruitment and NET formation in colitis mice.

**Figure 6:**
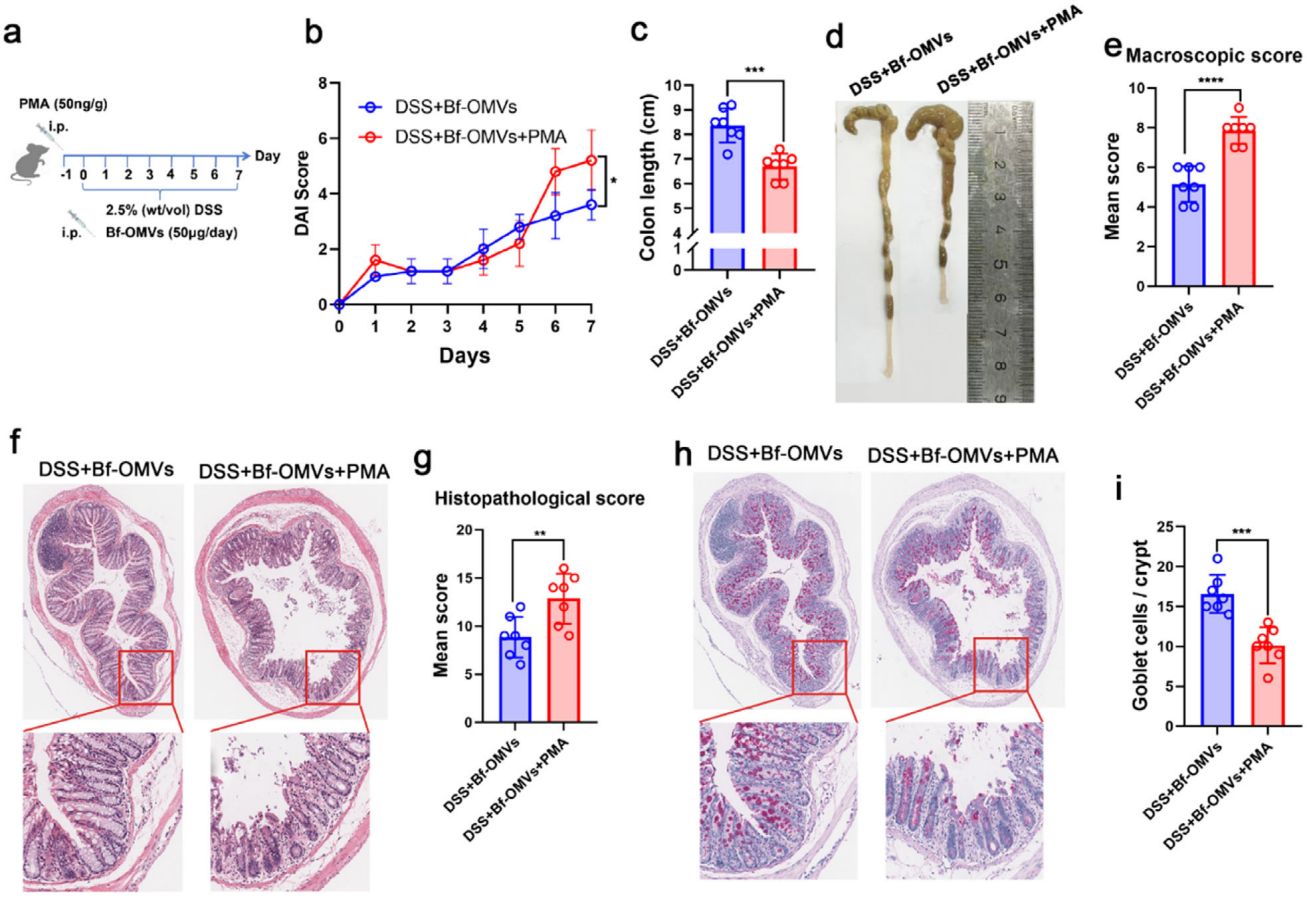
Bf‐OMVs inhibit NET formation to alleviate DSS‐induced colitis. a) Mice with DSS‐induced colitis were treated with Bf‐OMVs and PMA. b) DAI scores. c) Colon length measurements. d) Macroscopic appearance of the colon. e) Macroscopic colon scores. f) Histopathological examination of colon tissues stained with H&E. g) Histopathological scores for the colon tissue samples were determined by H&E staining. h, i) AB‐PAS staining used to assess goblet cell depletion, with goblet cell counts presented. n = 7; results are presented as the mean ± SD; ^*^ *p* < 0.05,^**^ *p* < 0.01, ^***^ *p* < 0.001, ^****^ *p* < 0.0001.

To determine whether Bf‐OMVs alleviate DSS‐induced colitis specifically through NET inhibition, we administered PMA to Bf‐OMV‐treated mice to artificially induce NET formation (**Figure** **6**a). Subsequent analyses revealed that PMA‐driven NETosis substantially diminished the protective effects of Bf‐OMVs, as evidenced by DAIs, colon lengths, macroscopic colon scores, histological analyses, histological scores, and goblet cell depletion (Figure 6b‐i). These data suggest that Bf‐OMVs ameliorate colitis by suppressing NET formation.

### 2.6 Bf‐OMVs Protect Mice Against DSS‐Induced Colitis by Carrying miR‐5119

**Figure 7:**
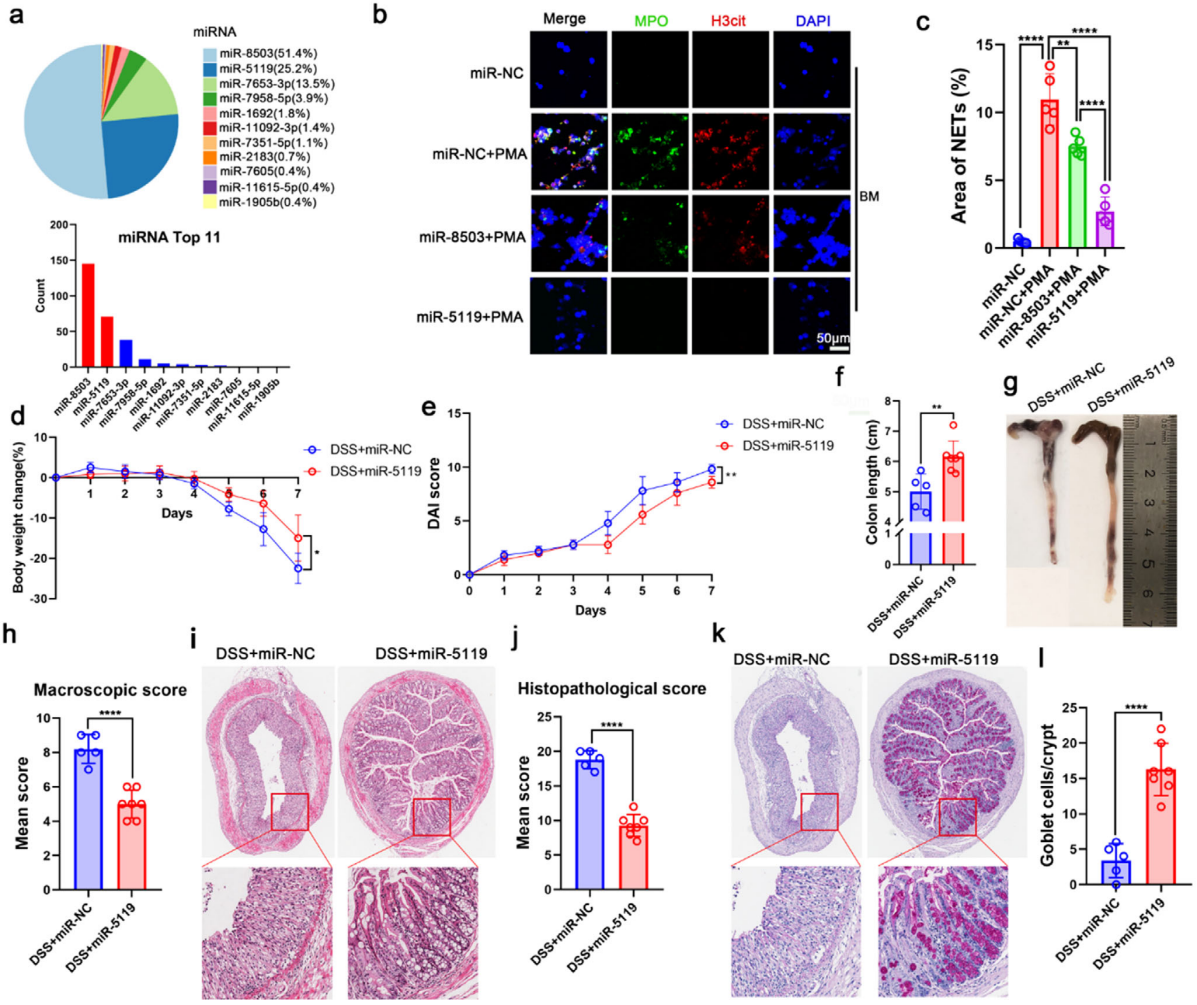
Bf‐OMVs protect mice against DSS‐induced colitis by carrying miR‐5119. a) The expression profile of sRNAs in Bf‐OMVs, sRNAs were compared with eukaryotes’ miRNAs sequences in the miRBase database, with the top 11 most highly expressed miRNAs presented. b, c) BM neutrophils were treated with miR‐8503 and miR‐5119 while NET formation was induced with PMA, NET formation was observed using immunofluorescence microscopy. d) Body weight loss during colitis progression was recorded. e) Changes in the DAI were assessed. f) On day 7, mice were euthanized, and their colons were excised and measured for length. g) The macroscopic appearance of the colons was recorded. h) Average macroscopic colon scores were calculated for each group. i) Histopathological changes in colon tissues were examined using H&E staining. j) Histopathological scoring was conducted for colon tissue samples. k, l) AB‐PAS staining was used to assess goblet cell depletion in the colon (k), with the quantification of goblet cells shown (l). n = 5–7; results are presented as the mean ± SD; ^*^ *p* < 0.05, ^**^ *p* < 0.01, ^****^ *p* < 0.0001.

OMVs are enriched in bacterial small RNAs (sRNAs).^[^ ^24^ ^]^ The bacterial sRNAs contained in OMVs exhibit sequence similarity to eukaryotic microRNAs (miRNAs) and can regulate host immune responses through targeted mechanisms.^[^ ^24^ ^]^ MiRNAs, which are major components carried by extracellular vesicles, play key roles in regulating gene expression.^[^ ^25^ ^]^ Therefore, we analyzed the expression profile of sRNAs in Bf‐OMVs. To predict their potential functions in host cells, we compared these sRNAs with eukaryotes’ miRNAs sequences in the miRBase database (http://www.mirbase.org/). Among the sRNAs carried by Bf‐OMVs, two highly abundant sRNAs were found to completely match the sequences of pxy‐miR‐8503 and mmu‐miR‐5119 (**Figure** **7**a). We then explored the potential roles of miR‐8503 and miR‐5119 in Bf‐OMV‐mediated NET inhibition. BM neutrophils were treated with miR‐8503 and miR‐5119 while NET formation was induced with PMA. Both miR‐8503 and miR‐5119 were found to inhibit PMA‐induced NET formation. Interestingly, miR‐5119 exhibited a more significant inhibitory effect on NET formation compared with miR‐8503 (Figure 7b,c).

To further examine the therapeutic effect of miR‐5119, we administered miR‐5119 to mice with colitis via intraperitoneal injection (Figure S9, Supporting Information). As shown in Figure 7d, body weight loss was significantly reduced in the DSS+miR‐5119 group compared with the DSS+miR‐NC group (miR‐NC, a universal negative control for the study of miRNA) from day 5 onward (Figure 7d). The DAI of the DSS+miR‐5119 group was consistently lower than that of the DSS+miR‐NC group from day 4 onward (Figure 7e). Additionally, the colon lengths of mice in the DSS+miR‐5119 group were significantly greater than those of mice in the DSS+miR‐NC group (Figure 7f,g). The DSS+miR‐5119 group also displayed significantly lower macroscopic colon scores and less colonic architecture disruption compared with the DSS+miR‐NC group (Figure 7h,i). Consistently, the histological scores were lower in miR‐5119‐treated mice with colitis (Figure 7j). Furthermore, the DSS‐induced goblet cell depletion was alleviated following miR‐5119 treatment (Figure 7k,l). These findings suggest that Bf‐OMVs protect against DSS‐induced colitis by delivering a high abundance of miR‐5119.

### 2.7 MiR‐5119 Delivery by Bf‐OMVs Inhibits Neutrophil Recruitment and NET Formation in Mice with Colitis

**Figure 8:**
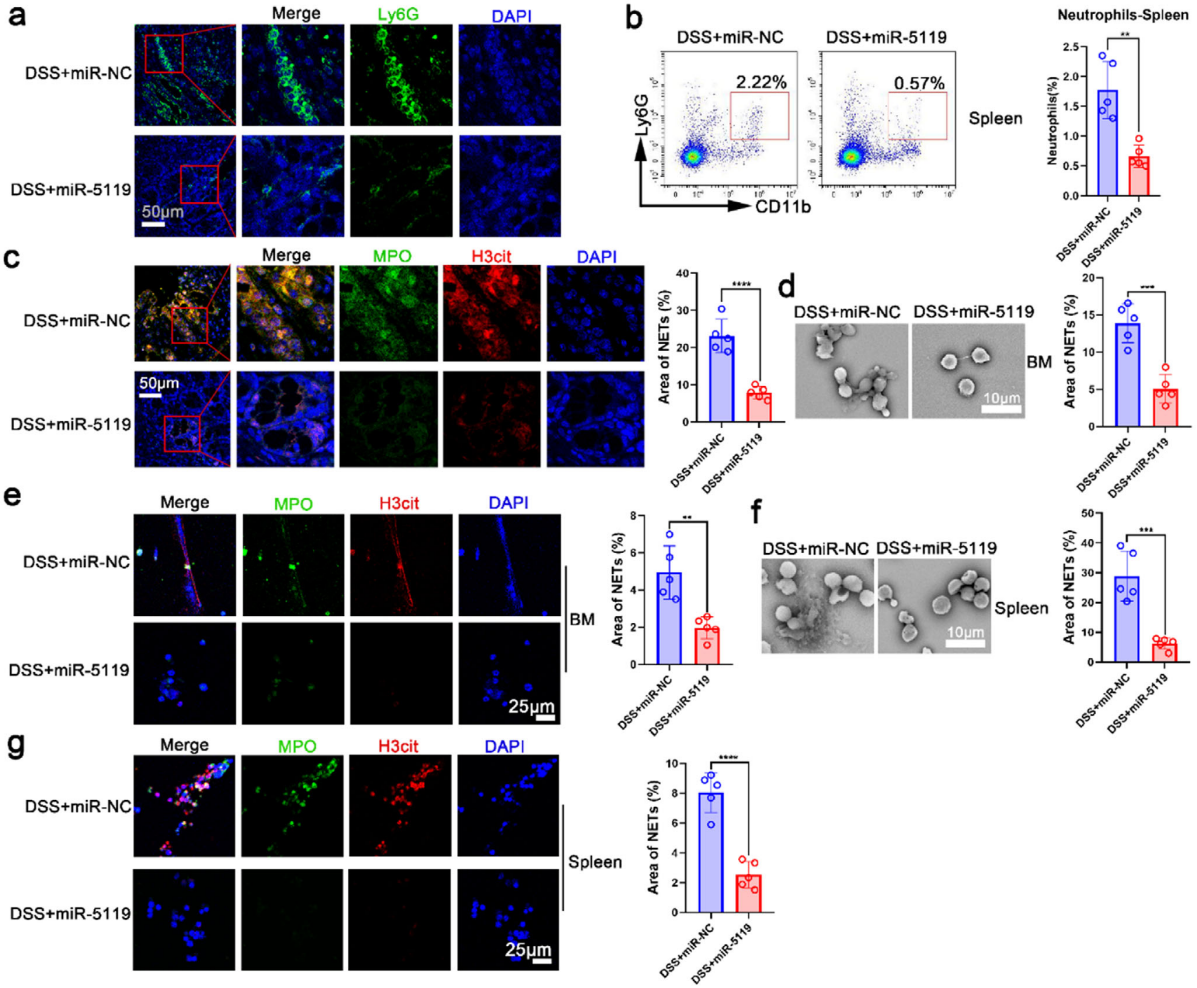
MiR‐5119 delivery by Bf‐OMVs inhibits neutrophil recruitment and NET formation in mice with colitis. a) Immunofluorescence analyses were performed to assess neutrophil levels in colon tissues. b) The splenic neutrophil percentage was determined using flow cytometry, with statistical analysis results displayed. c) Immunofluorescence was used to evaluate NET formation in the colon tissues of mice with colitis. d–g) Neutrophils isolated from the BMs and spleens of mice were cultured in vitro for 24 h. SEM and immunofluorescence analyses were performed to assess NET formation. n = 5–7; results are presented as the mean ± SD; ^*^ *p* < 0.05, ^**^ *p* < 0.01, ^***^ *p* < 0.001, ^****^ *p* < 0.0001.

To assess whether miR‐5119 plays a crucial role in Bf‐OMVs inhibiting neutrophil infiltration and NET formation to alleviate colitis, we evaluated neutrophil levels in the intestinal mucosa and spleens of treated mice. A significant reduction in neutrophil infiltration in the intestinal mucosa was observed following miR‐5119 treatment (**Figure** **8**a). Additionally, the percentage of neutrophils in the spleens of miR‐5119‐treated mice was significantly lower than that in miR‐NC‐treated mice with colitis (Figure 8b). Moreover, miR‐5119 treatment significantly reduced NET formation in the intestinal mucosa of mice with colitis compared with miR‐NC treatment (Figure 8c). We further examined neutrophils from the BM and spleen of mice with colitis after 24‐h in vitro culture and observed that miR‐5119 treatment significantly downregulated NET formation (Figure 8d–g). Compared with miR‐NC‐treated mice with colitis, neutrophils from miR‐5119‐treated mice formed fewer NETs when stimulated by PMA (Figure S10a–d, Supporting Information). These results indicate that miR‐5119 delivered by Bf‐OMVs inhibits neutrophil recruitment and NET formation in mice with colitis.

### 2.8 MiR‐5119 Inhibits NET Formation by Targeting PD‐L1 to Downregulate GSDMD‐Mediated NETs Release, thus Alleviating DSS‐Induced Colitis

**Figure 9:**
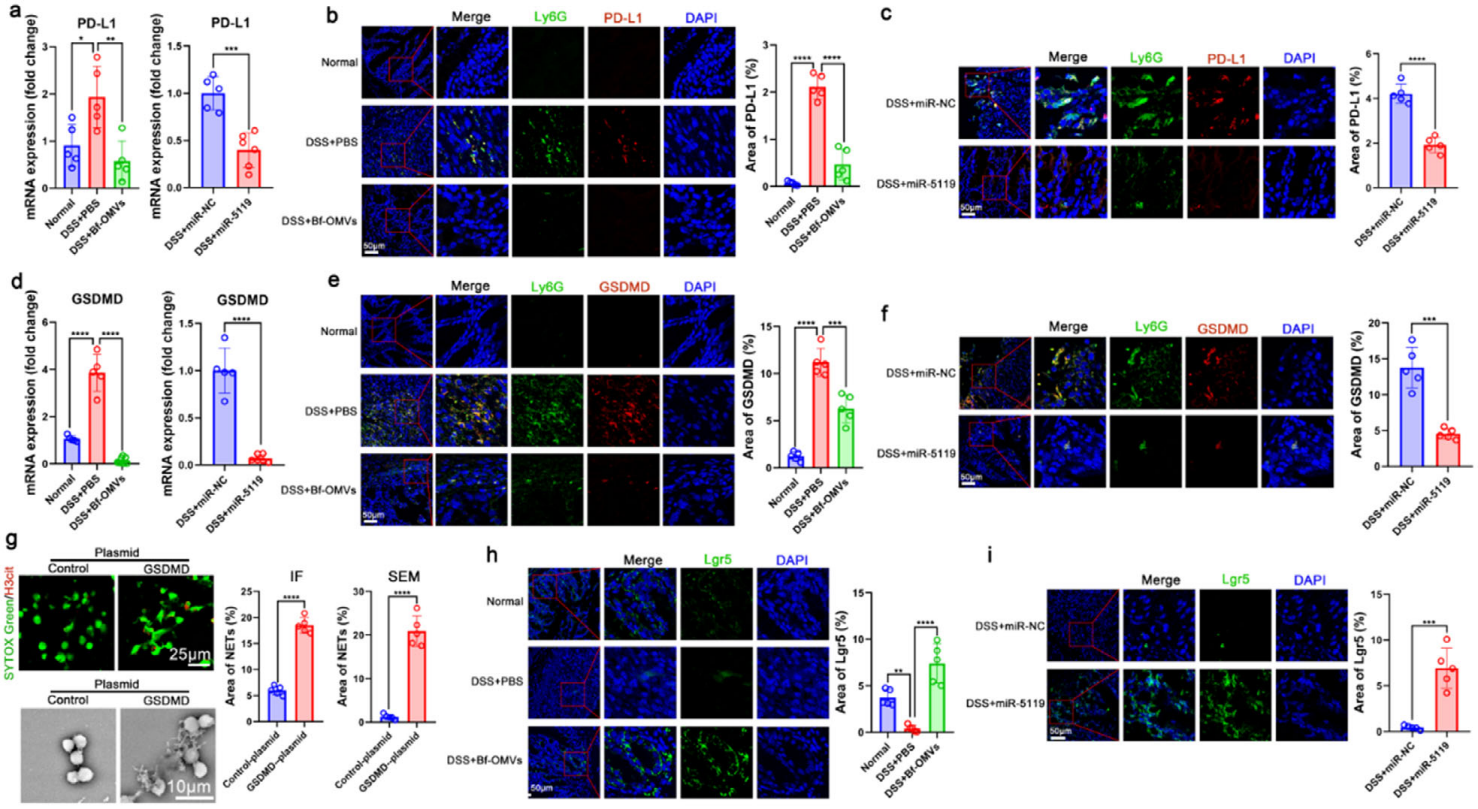
MiR‐5119 inhibits NET formation by targeting PD‐L1 to downregulate GSDMD‐mediated NET release. a) The mRNA expression of PD‐L1 was measured using qRT‐PCR. b,c) Immunofluorescence analyses were performed to detect PD‐L1 expression in neutrophils in the colon, and the fluorescence area of PD‐L1 expression was statistically quantified. d) The mRNA expression of GSDMD was measured using qRT‐PCR. e,f) Immunofluorescence analyses were performed to detect GSDMD expression in neutrophils in the colon, and the fluorescence area of GSDMD expression was statistically quantified. g) Neutrophils were transfected with GSDMD overexpression plasmid or negative control plasmid. NETs were assessed by immunofluorescence (SYTOX Green and H3cit co‐staining) and SEM. h, i) Lgr5^+^ cells in the intestinal mucosa were detected by immunofluorescence to assess intestinal stem cell (ISC) levels. n = 5–6; results are presented as the mean ± SD; ^*^ *p* < 0.05, ^**^ *p* < 0.01, ^***^ *p* < 0.001, ^****^ *p* < 0.0001.

We further elucidate the molecular mechanism by which miR‐5119 inhibits NET formation. Studies have shown that PD‐L1 promotes Gasdermin D (GSDMD)‐mediated NET release.^[^ ^26^ ^]^ We found that the colons of mice with colitis expressed high levels of PD‐L1, while treatment with Bf‐OMVs significantly downregulated PD‐L1 expression (**Figure** **9**a). MiR‐5119 was found to directly target PD‐L1 and inhibit its expression (Figure S11, Supporting Information).^[^ ^27^ ^]^ Compared to colitis mice treated with miR‐NC, those treated with miR‐5119 showed a significant reduction in PD‐L1 expression in the colons (Figure 9a). Neutrophils in the colons of colitis mice expressed high levels of PD‐L1, while treated with Bf‐OMVs and miR‐5119 showed significant downregulation of PD‐L1 expression (Figure 9b,c). Similarly, neutrophils in the colons of mice with colitis treated with *B. fragilis* and Bf^OMVs+^ showed significant downregulation of PD‐L1 expression, with the Bf^OMVs+^ group exhibiting a more pronounced effect (Figure S12a, Supporting Information). In contrast, the downregulation of PD‐L1 expression in the Bf^OMVs−^group was not significant (Figure S12a, Supporting Information).

Moreover, we observed that colonic tissues of mice with colitis had elevated levels of GSDMD expression, which was significantly diminished in mice treated with Bf‐OMVs and miR‐5119 (Figure 9d). Neutrophils in the colons of mice with colitis expressed high levels of GSDMD, which were reduced following treatment with Bf‐OMVs and miR‐5119 (Figure 9e,f). Similarly, neutrophils in the colons of mice with colitis treated with *B. fragilis* and Bf^OMVs+^ displayed significant downregulation of GSDMD expression, with the Bf^OMVs+^ group showing a more pronounced effect (Figure S12b, Supporting Information). In contrast, the reduction in GSDMD expression in the Bf^OMVs−^group was not significant (Figure S12b, Supporting Information). We constructed GSDMD overexpression plasmids and transfected them into neutrophils (Figure S12c, Supporting Information), finding that GSDMD overexpression significantly promoted NET formation (Figure 9g), suggesting miR‐5119 inhibits NETs by downregulating GSDMD.

Dysfunction of intestinal stem cells (ISCs) is a hallmark of IBD, and reducing *Enterococcus*/tyramine‐mediated suppression of ISCs alleviates colitis.^[^ ^28^ ^]^ ISCs are a promising therapeutic target for IBD.^[^ ^29^ ^]^ In mice with colitis, ISCs (Lgr5^+^ cells) in the intestinal mucosa were significantly reduced compared with normal controls; however, their levels were significantly upregulated following treatment with Bf‐OMVs and miR‐5119 (Figure 9h,i). Similarly, the number of ISCs in the colon tissues of mice with colitis treated with *B. fragilis* and Bf^OMVs+^ was increased, with the Bf^OMVs+^ group showing a more pronounced increase (Figure S12d, Supporting Information). In contrast, the increase in ISCs in the Bf^OMVs−^group was less significant (Figure S12d, Supporting Information).

**Figure 10:**
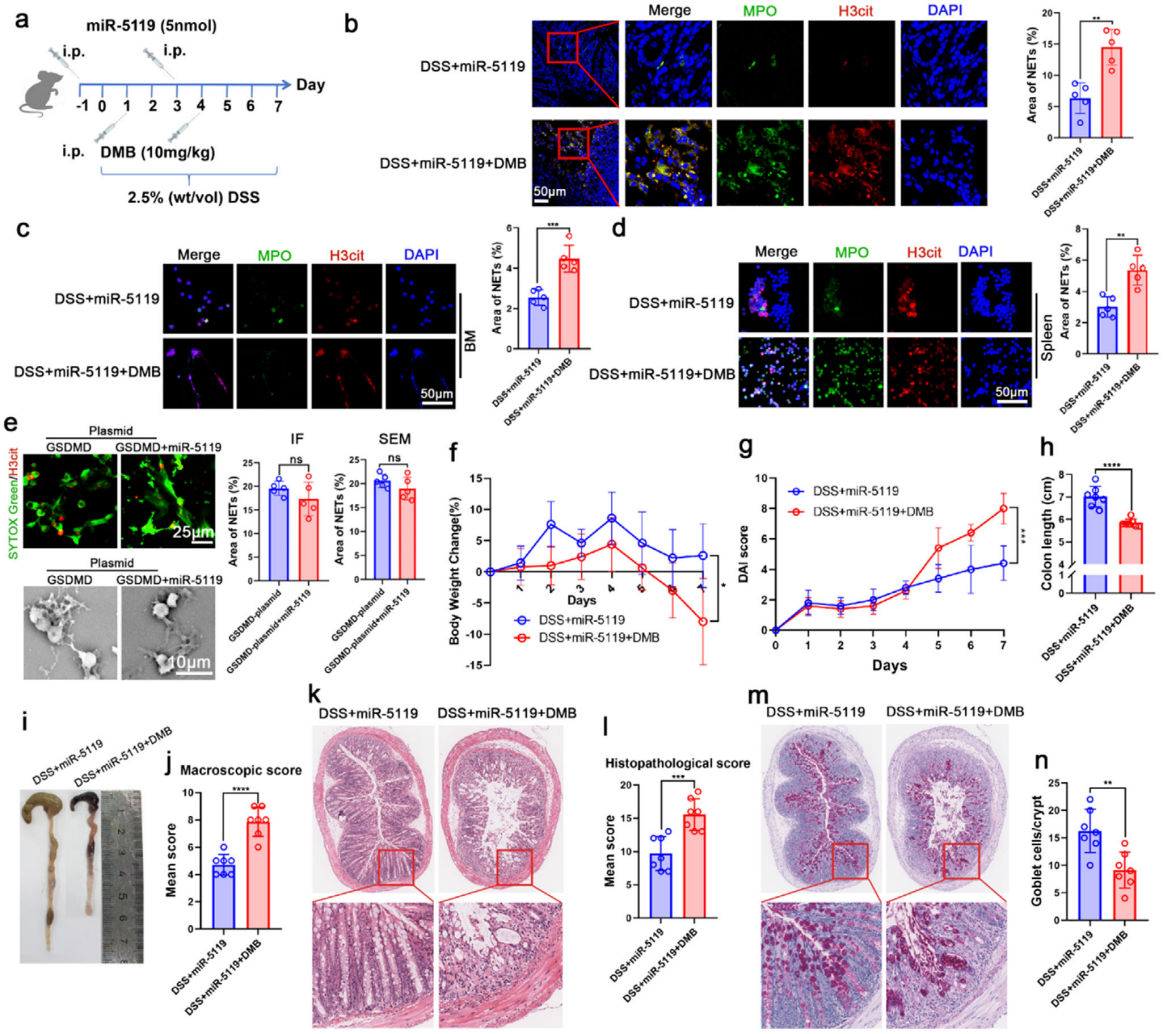
MiR‐5119 inhibits NET formation by targeting PD‐L1 to downregulate GSDMD‐mediated NETs release, thus alleviating DSS‐induced colitis. a) Mice with DSS‐induced colitis were treated with miR‐5119 and DMB. b) Immunofluorescence was used to evaluate NET formation in the colon tissues of mice with colitis. c, d) Neutrophils isolated from the BMs and spleens of mice were cultured in vitro for 24 h. Immunofluorescence analysis was performed to assess NET formation. e) Neutrophils were treated with miR‐5119 while using GSDMD overexpression plasmid to induce NET formation in vitro; NETs were assessed by immunofluorescence (SYTOX Green and H3cit co‐staining) and SEM. f) Body weight loss during colitis progression was recorded. g) DAI scores. h) Colon length measurements. i) Macroscopic appearance of the colon. j) Macroscopic colon scores. k) Histopathological examination of colon tissues stained with H&E. l) Histopathological scores for the colon tissue samples were determined by H&E staining. m, n) AB‐PAS staining used to assess goblet cell depletion, with goblet cell counts presented. n = 5–7; results are presented as the mean ± SD; ^*^ *p* < 0.05,^**^ *p* < 0.01, ^***^ *p* < 0.001, ^****^ *p* < 0.0001.

To validate the role of GSDMD in miR‐5119‐mediated NET suppression, we administered 6,7‐dichloro‐2‐methylsulfonyl‐3‐N‐tert‐butylaminoquinoxaline (DMB), a selective GSDMD agonist,^[^ ^30^ ^]^ to miR‐5119‐treated mice (**Figure** **10**a). DMB‐driven GSDMD activation and GSDMD overexpression with plasmids substantially attenuated miR‐5119′s inhibitory effects on NET formation (Figure 10b–e), and compromised its therapeutic efficacy in alleviating colitis (Figure 10f–n).

These results suggest that miR‐5119 inhibits NET formation by targeting PD‐L1 to downregulate GSDMD‐mediated NET release, thereby alleviating DSS‐induced colitis.

## 3 Discussion

*Bacteroides fragilis* is a common resident of the human gut, and studies have shown that it can alleviate colitis by modulating host immune responses.^[^ ^4^, ^7^, ^8^, ^9^ ^]^ However, the exact mechanisms by which *B. fragilis* exerts its anti‐inflammatory effects have not been fully elucidated. In this study, we demonstrated that promoting *B. fragilis* OMV secretion enhances its ability to alleviate colitis while inhibiting OMV secretion reduces this effect. Furthermore, we found that OMVs can alleviate colitis by inhibiting NET formation. Specifically, Bf‐OMVs are enriched with miR‐5119, which targets and inhibits PD‐L1, leading to the suppression of GSDMD‐mediated NET release and then colitis alleviation. These findings provide new insights into the role of *B. fragilis* OMVs in the pathogenesis and treatment of IBD.

In the intestinal ecosystem, the bilateral communication between microbes and the host is rarely mediated by direct cell‐to‐cell contact due to the presence of the epithelial barrier.^[^ ^31^ ^]^ Gram‐negative bacteria can release OMVs to protect various parental components, such as nucleic acids, proteins, enzymes, and lipopolysaccharides, enabling their long‐distance transport. Therefore, OMVs play a crucial role in microbe‐host interactions. Accumulating evidence shows that microbiota‐derived OMVs modulate immune responses by carrying molecules that target immune cells, particularly macrophages and neutrophils.^[^ ^32^, ^33^ ^]^ *Fusobacterium nucleatum* aggravates rheumatoid arthritis through FadA‐containing OMVs that trigger synovial macrophages.^[^ ^34^ ^]^ *Helicobacter pylori*‐derived OMVs contribute to Alzheimer's disease pathogenesis by activating glial cells and inducing neuronal dysfunction.^[^ ^35^ ^]^ OMVs released from *Akkermansia muciniphila* are able to elicit a mucosal immunoglobulin A response by translocating into Peyer's patches and subsequently activating B cells and dendritic cells.^[^ ^11^ ^]^ Additionally, OMVs from probiotics can alleviate disease progression. *Lactobacillus plantarum*‐derived OMVs induce anti‐inflammatory M2 macrophage polarization in vitro.^[^ ^36^ ^]^ L*actobacillus murinus*‐derived OMVs TLR2 promotes M2 macrophage polarization and releases IL‐10, thereby alleviating deoxynivalenol‐induced intestinal barrier disruption.^[^ ^37^ ^]^ *Lactobacillus plantarum*‐derived OMVs modulate macrophage polarization and gut homeostasis to alleviate ulcerative colitis.^[^ ^38^ ^]^ *Clostridium butyricum*‐derived OMVs also modulate gut homeostasis and ameliorate acute experimental colitis.^[^ ^39^ ^]^ Many studies have reported that *B. fragilis* can alleviate colitis; however, the underlying mechanisms have not been fully elucidated. Here, we demonstrated that enhancing *B. fragilis* OMV secretion augments its therapeutic efficacy, whereas suppressing OMV production diminishes this effect. Notably, intraperitoneal injection or oral administration of purified Bf‐OMVs significantly alleviated DSS‐induced colitis in mice, indicating that OMVs are essential mediators of *B. fragilis*’s anti‐inflammatory function.

NETs protect against infections, particularly those caused by large pathogens; however, they are also implicated in the pathogenes of a growing number of immune‐mediated conditions.^[^ ^40^ ^]^ NETs have been proposed as a source of self‐antigens in autoimmune diseases, especially those associated with autoantibodies directed against neutrophil‐derived proteins.^[^ ^40^ ^]^ NETs are associated with inflammatory diseases and are thought to maintain mucosal inflammation in IBD.^[^ ^19^ ^]^ NET‐associated histones alter the integrity of tight junctions and adhere to junctional proteins, inducing intestinal epithelial cell (IEC) death and increasing intestinal epithelial permeability.^[^ ^41^ ^]^ Therapies targeting NET formation can alleviate colitis. Cyclosporine A alleviates colitis by inhibiting the formation of NETs via the regulation of the pentose phosphate pathway.^[^ ^42^ ^]^ Knocking out PAD4 to block NET formation reduces clinical colitis indices, intestinal inflammation, and barrier dysfunction.^[^ ^43^ ^]^ In this study, we found that Bf‐OMVs significantly inhibit the recruitment of neutrophils from the bone marrow to the periphery and downregulate neutrophil infiltration in the colon tissues. Furthermore, Bf‐OMVs directly target neutrophils and inhibit NET formation. To confirm the dependency of Bf‐OMVs’ therapeutic effects on NET inhibition, we artificially induced NETosis using phorbol myristate acetate (PMA) in Bf‐OMV‐treated mice. Remarkably, PMA‐driven NETosis substantially diminished the protective effects of Bf‐OMVs, underscoring that NET suppression is central to Bf‐OMV‐mediated colitis alleviation.

OMVs act as carriers for bioactive molecules, including nucleic acids, proteins, and lipids, enabling remote modulation of host pathways. Bacterial sRNAs within OMVs share homology with host microRNAs and regulate immune responses.^[^ ^24^ ^]^ We identified a highly abundant mmu‐miR‐like sRNA, miR‐5119, in Bf‐OMVs that potently protects against DSS‐induced colitis. GSDMD is a pore‐forming protein and an executor of pyroptosis. A previous study has shown that GSDMD plays a vital role in NET generation.^[^ ^44^ ^]^ GSDMD drives canonical inflammasome‐induced neutrophil pyroptosis and is dispensable for NETosis.^[^ ^45^ ^]^ GSDMD cleavage by human caspase‐4/5 or mouse caspase‐11 creates pores in neutrophil granule membranes, allowing the release of neutrophil elastase (NE) and myeloperoxidase (MPO) during the initial stages of NET formation. Later stages of NET formation also require GSDMD activation, which is responsible for the formation of pores in the cell membrane that allow NETs extrusion.^[^ ^46^ ^]^ GSDMD promotes NET formation via the mtDNA‐cGAS‐STING pathway during lung ischemia/reperfusion.^[^ ^47^ ^]^ Neutrophil GSDMD‐mediated NETs contribute to sepsis‐related lung, heart, kidney, and liver injury, and inhibiting GSDMD reverses the sepsis‐induced damage in these organs.^[^ ^46^ ^]^ A previous study revealed that PD‐L1 can promote GSDMD‐mediated NET release by maintaining the transcriptional activity of STAT3 in sepsis‐associated encephalopathy.^[^ ^26^ ^]^ PD‐L1 is a direct target of miR‐5119.^[^ ^27^ ^]^ We found that miR‐5119 inhibits NET formation by targeting PD‐L1 to downregulate GSDMD. Studies indicate that impaired ISC function in patients with CD and mice models is responsible for its chronic, relapsing inflammation.^[^ ^29^, ^48^, ^49^ ^]^ Interestingly, we observed that in mice with colitis, ISC levels in the intestinal mucosa were significantly reduced compared with normal controls; however, their levels were significantly upregulated following treatment with Bf‐OMVs and miR‐5119. This suggests that NETs may inhibit ISC proliferation, while Bf‐OMVs and miR‐5119 can promote their proliferation, thereby facilitating the differentiation of ISCs into intestinal epithelial cells and repairing colonic damage. However, further research is needed to confirm this hypothesis.

To validate GSDMD's role in miR‐5119‐mediated NET suppression, we administered the GSDMD agonist DMB to miR‐5119‐treated mice. DMB‐driven GSDMD activation substantially attenuated miR‐5119′s inhibitory effects on NET formation and attenuated its therapeutic efficacy in colitis. Confirming that miR‐5119 inhibits NET formation by targeting PD‐L1 to downregulate GSDMD‐mediated NET release, thereby alleviating DSS‐induced colitis.

In summary, our study demonstrated that *B. fragilis* alleviates DSS‐induced colitis through the secretion of OMVs, which inhibit NET formation. Intraperitoneal injection of Bf‐OMVs and the high abundance of miR‐5119 in these vesicles effectively alleviate colitis by inhibiting NET formation. This study provides new insights into the mechanisms by which *B. fragilis* can alleviate colitis and suggests a potential therapeutic avenue for IBD.

## 4 Experimental Section

### Animals and Ethics

Male BALB/c mice (six weeks old) were procured from the Guangdong Medical Laboratory Animal Center, China. All animal procedures and experimental protocols were approved by the Animal Care and Use Committee of Guangzhou Medical University (approval number: G2023‐726) and conformed to the Guidelines for the Care and Use of Laboratory Animals of the National Institute of Health in China.

### Bacterial Strains and Culture Preparation

The non‐toxigenic strain of *Bacteroides fragilis* ATCC25285 was procured from the American Type Culture Collection (ATCC; located in Manassas, VA, USA). This strain was cultivated anaerobically in Brain Heart Infusion (BHI) broth at 37 °C, within an anaerobic atmosphere consisting of 5% H_2_, 10% CO_2_, and 85% N_2_, for 48 h. To either stimulate or hinder the secretion of OMVs by *B. fragilis*, the bacteria were subjected to treatment with either monensin (an OMV secretion‐promoting agent, 7 µm, MCE) or GW4869 (an OMV secretion‐inhibiting agent, 20 µm, MCE) for 24 h.^[^ ^20^, ^21^ ^]^ Then, *B. fragilis* was centrifuged, rinsed thrice with sterile PBS solution, and finally adjusted to a concentration of 1 × 10^9^ CFU mL^−1^ in sterile PBS by gauging the optical density at 600 nm using a spectrophotometer and by quantifying CFUs after plating on blood agar.

### Isolation and Characterization of *B. fragilis* OMVs

*B. fragilis* was anaerobically cultured in BHI broth at 37 °C in an atmosphere consisting of 5% H_2_, 10% CO_2_, and 85% N_2_ for 72 h. The bacteria were then subjected to sequential centrifugation steps: 1000 g for 10 min, 3000 g for 20 min, and 10 000 g for 30 min, to remove debris and cells. The resulting supernatant was filtered through a 0.22 µm membrane and subsequently ultracentrifuged at 120 000 g for 90 min using an Optima L‐100xp ultracentrifuge (Beckman Coulter, USA). The pellet was resuspended in PBS, and the OMVs were quantified based on their protein content using a BCA protein assay kit (Beyotime, China). The samples were stored at −80 °C until further use.

The morphological analysis of OMVs was performed using negative‐staining transmission electron microscopy (TEM). Briefly, OMVs were placed on a copper grid and stained with 3% (w/v) aqueous phosphotungstic acid for one min before examination with a JEM‐1400PLUS TEM (Jeol, Japan). Additionally, OMVs were characterized using High‐Sensitivity Flow Cytometry for Nanoparticle Analysis (N30, NanoFCM, China).

### Induction of DSS Colitis and Treatment

Acute colitis was induced by administering 2.5% (wt/vol) dextran sulfate sodium (DSS, molecular weight 36–50 kDa, MP Biomedicals) in the drinking water for eight days (from day 0 to day 7). Mice with colitis were then treated with one of the following: *B. fragilis* (gavage, 1 × 10^8^ CFU), *B. fragilis* that promotes OMV secretion (Bf^OMVs+^) (gavage, 1 × 10^8^ CFU), *B. fragilis* that inhibits OMV secretion (Bf^OMVs−^) (gavage, 1 × 10^8^ CFU), OMVs (intraperitoneal injection, 50 µg), OMVs (gavage, 2 × 10^10^ vesicles), PMA (intraperitoneal injection, 50 ng g^−1^), miR‐NC (intraperitoneal injection, 5 nmol), miR‐NC (intraperitoneal injection, 5 nmol), or DMB (intraperitoneal injection, 10 mg kg^−1^). Control groups (Normal and DSS) received equivalent volumes of PBS.

### Clinical and Histological Evaluation of Colitis

Colitis progression was monitored daily by tracking changes in body weight, stool consistency, and the presence of fecal blood, which was detected using a Hemoccult assay kit (Nanjing Jiancheng Bio‐engineering Institute, China). The Disease Activity Index (DAI) was calculated based on these observations in accordance with established protocols.^[^ ^50^ ^]^

On day 7, the mice were euthanized, and their colon lengths were measured. A macroscopic examination of the colons was conducted by an investigator blinded to the experimental groups. The evaluation considered factors including hyperemia, colon wall thickness, ulceration, the extent of inflammation, and overall tissue damage.^[^ ^50^ ^]^ Colonic tissue samples were then collected, fixed in 4% paraformaldehyde, processed, and embedded in paraffin. The tissue sections were stained with hematoxylin and eosin (H&E) for histopathological analysis. The histopathological scores of the colonic lesions were determined based on inflammation severity, immune cell infiltration, crypt damage, submucosal edema, goblet cell loss, and epithelial hyperplasia.^[^ ^50^ ^]^

### Goblet Cell Quantification

Alcian blue‐periodic acid Schiff (AB‐PAS) staining was used to visualize and quantify goblet cells in colonic sections. The number of goblet cells per crypt was then determined.

### Flow Cytometric Analysis of Neutrophil Subsets

Neutrophil subsets were analyzed in cells isolated from the colon, spleens, and femoral bone marrow. Single‐cell suspensions were incubated for 20 min at room temperature with Zombie (BioLegend, USA). Cells were then washed and stained with fluorochrome‐conjugated monoclonal antibodies against CD45, CD11b, and Ly6G (BioLegend, USA) per the manufacturer's instructions. After 20 min, cells were washed, resuspended in PBS, and analyzed on a CytoFLEX S flow cytometer (Beckman Coulter, USA). Neutrophils were identified as Zombie negative (live cells), CD45^+^CD11b^+^Ly6G^+^ cells. Neutrophil populations were quantified with CytExpert software (Beckman Coulter, USA).

### Neutrophil Isolation and In Vitro Treatments

Neutrophils were isolated from the mouse bone marrow (femur and tibia) and spleens using Percoll density gradient centrifugation. The isolated neutrophils were cultured at 37 °C in a humidified atmosphere with 5% CO_2_. For in vitro experiments, neutrophils were seeded on coverslips and treated with Bf, Bf^OMVs+^ (1 × 10^5^ CFU mL^−1^), Bf^OMVs−^(1 × 10^5^ CFU mL^−1^), Bf‐OMVs (20 µg mL^−1^), Bf‐OMVs‐free (20 µg mL^−1^), TNF‐α (20 ng mL^−1^, MCE, China), miR‐NC (20 µm), or miR‐5119 (20 µm) for 24 h. PMA (150 nM) was added four h before cell collection to stimulate NET formation.

### Immunofluorescence Analysis

For immunofluorescence examinations of colon tissue sections, specimens were fixed, embedded in paraffin, and sectioned. The sections were then deparaffinized, rehydrated, and blocked with 1% BSA. Subsequently, the samples were incubated overnight at 4 °C with the following primary antibodies: anti‐Ly6G (1:100; Abcam, UK), anti‐myeloperoxidase (MPO, 1:100; Abcam, UK), anti‐H3cit (1:200; CST, USA), anti‐gasdermin D (GSDMD, 1:100; Proteintech, China), and anti‐Lgr5 (1:200; Abcam, UK). For cultured cell analyses, the cells were fixed with 4% paraformaldehyde for 25 min at room temperature, permeabilized with 0.1% Triton X‐100 in PBS for 4 min, and blocked with PBS containing 2% BSA for 30 min at room temperature. The cells were then incubated overnight at 4 °C with anti‐MPO (1:100, Abcam, UK) and anti‐H3Cit (1:200, CST, USA). Both tissue sections and cultured cells were subsequently treated with appropriate Alexa Fluor‐conjugated secondary antibodies. Nuclei were stained with DAPI or SYTOX Green. Throughout the procedure, samples were washed thrice in PBS. Visualization was performed using a TCS SP8 DLS laser scanning confocal microscope (Leica, Germany). The NETs were determined as the percentage of the positive H3cit signal. For each section, at least 10 randomly selected fields were analyzed using ImageJ software. The percentage of NETs per section was calculated as the mean value across these fields (≥ 10 fields/section), with n = 5 per group.

### Scanning Electron Microscopy

Neutrophils cultured on coverslips were fixed in 2.5% glutaraldehyde overnight. After fixation, the samples were washed with PBS and then dehydrated through an ethanol gradient. This was followed by the replacement of ethanol with acetone and isoamyl acetate. The specimens were subsequently subjected to critical point drying and gold coating using an ion coater. Observation and imaging were conducted using a TM4000Plus II scanning electron microscope (HITACHI, Japan).

### Analysis of OMVs Uptake

OMVs were labeled with the PKH26 fluorescent dye (MCE, China) for 5 min. To stop the staining process, an equal volume of 1% BSA was added. The labeled OMVs were then transferred to a Quick‐Seal centrifuge tube (Beckman Coulter, USA) and subjected to ultracentrifugation at 120 000 g for 90 min at 4 °C using an Optima L‐100XP ultracentrifuge with a swinging‐bucket rotor (model SW60 Ti, Beckman Coulter). The resulting pellet, which contained the PKH26‐labeled OMVs, was resuspended in PBS and subjected to a second round of ultracentrifugation under the same conditions to ensure purity. To evaluate the internalization of OMVs, neutrophils were incubated with the purified PKH26‐labeled OMVs for 1 h. The cells were then prepared for confocal microscopy analysis. Actin was labeled using Alexa Fluor phalloidin‐FITC (CST, USA), and nuclei were stained with DAPI.

### Plasmid Constructs and Transfection

The overexpression plasmids of TNF‐α and GSDMD were designed and synthesized by Genechem (Shanghai, China). Gene transfections were performed using Lipofectamine 2000 Transfection Reagent (Thermo Fisher, USA) according to the manufacturer's protocols.

### In Vivo Biodistribution of OMVs

To investigate the in vivo biodistribution of OMVs, the OMVs were labeled with the DiR fluorescent dye (UElandy, China) by incubation at 37 °C for 30 min per the manufacturer's instructions. The labeled solution was then subjected to ultracentrifugation at 120 000 g for 90 min at 4 °C, and the resulting pellet was resuspended in 200 µL of PBS to obtain DiR‐Bf‐OMVs. Mice were intraperitoneally injected with 150 µg of DiR‐Bf‐OMVs per mouse and were euthanized 24 h post‐injection. The intestine, liver, kidneys, brain, heart, spleen, and femur were collected for ex vivo imaging analyses to monitor the biodistribution of the DiR‐Bf‐OMVs. DiR fluorescent signals were detected using the Odyssey CLx imaging system (LI‐COR Biosciences, USA).

### Small RNA Sequencing and Analysis

Small RNA sequencing and analyses were performed as previously described.^[^ ^51^ ^]^ Briefly, total RNA was isolated from OMVs and utilized as the input material for small RNA library construction. Following cluster generation, the library preparations were sequenced using the Illumina NovaSeq 6000 platform (Illumina, USA). After sequencing, the data underwent initial analyses, including quality control evaluation, comparative analysis, target gene functional annotation, and miRNA expression level quantification.

### RNA Extraction and Quantitative RT‐PCR (qRT‐PCR)

qRT‐PCR was employed to quantify mRNA expression. Specifically, RNA was extracted from cells by homogenizing 100 mg of colon tissues in TRIzol reagent (Invitrogen, USA), adhering to the manufacturer's instructions. The RNA yield was quantified using a NanoDrop ND‐2000 spectrophotometer (Thermo Scientific, USA). Complementary DNA (cDNA) was synthesized from 1.0 µg of total RNA using oligo(dT) primers and the Thermo Scientific RevertAid First Strand cDNA Synthesis Kit (Thermo Scientific), following the manufacturer's protocol. The expression levels of PD‐L1 and GSDMD were analyzed using a SYBR Green Master Mix kit (Takara, Japan), with primers outlined in **Table** **1**. GAPDH was used as an internal control, and the fold change in expression was calculated using the 2^‐ΔΔCT^ method.

**Table 1:** Primers used for qRT‐PCR.

Gene	Forward (5′‐3′)	Reverse (5′‐3′)
PD‐L1	GCTCCAAAGGACTTGTACGTG	TGATCTGAAGGGCAGCATTTC
GSDMD	TACTGCCTTCTGAACAGGAA	GTCACCACAAACAGGTCATC
GAPDH	ACTCCACTCACGGCAAATTC	TCTCCATGGTGGTGAAGACA

### Statistical Analysis

Results were expressed as mean ± SD. Comparisons between the two groups were conducted using unpaired two‐sample *t*‐tests. For multiple comparisons involving more than two groups, one‐way ANOVA was utilized. *P* values less than 0.05 were considered statistically significant. Immunofluorescence and scanning electron microscopy data were quantified using ImageJ software. All statistical analyses were performed using GraphPad Prism 8.0.

### Ethics Approval

All animal procedures and experimental protocols were approved by the Animal Care and Use Committee of Guangzhou Medical University (approval number: G2023‐726) and conformed to the Guidelines for the Care and Use of Laboratory Animals of the National Institute of Health in China.

## Conflict of Interest

The authors declare no conflict of interest.

## Author Contributions

Y.Y. designed the study, analyzed the data, and contributed to the writing of the manuscript. L.Y. performed the experiments. Y.Y. and H.D. assisted with the experimental procedures and data analysis. S.S. contributed to the interpretation of the results and provided feedback on the manuscript. Y.X. was involved in the collection and assembly of data and helped draft the manuscript. J.S. provided administrative support and contributed to the experimental design. Y.L., J.W., and J.Z. assisted with the statistical analysis of the data. Y.L. conceived the study, participated in its design and coordination, and helped to draft the manuscript. L.W. provided financial support for the research, contributed to the interpretation of the data, and helped to finalize the manuscript. All authors read and approved the final manuscript.

## Supporting information



Supporting Information

## Data Availability

The data that support the findings of this study are available from the corresponding author upon reasonable request.
