# Supplementary material for: Bacteroides Fragilis‐Derived Outer Membrane Vesicles Deliver MiR‐5119 and Alleviate Colitis by Targeting PD‐L1 to Inhibit GSDMD‐Mediated Neutrophil Extracellular Trap Formation
Source: Adv Sci (Weinh). 2025 Jun 25;12(35):e00781. doi: 10.1002/advs.202500781 (PMC12462929; doi:10.1002/advs.202500781)
Supplement: Supplementary file 1 — Supporting Information [file ADVS-12-e00781-s001.doc]

**Supplementary Information**


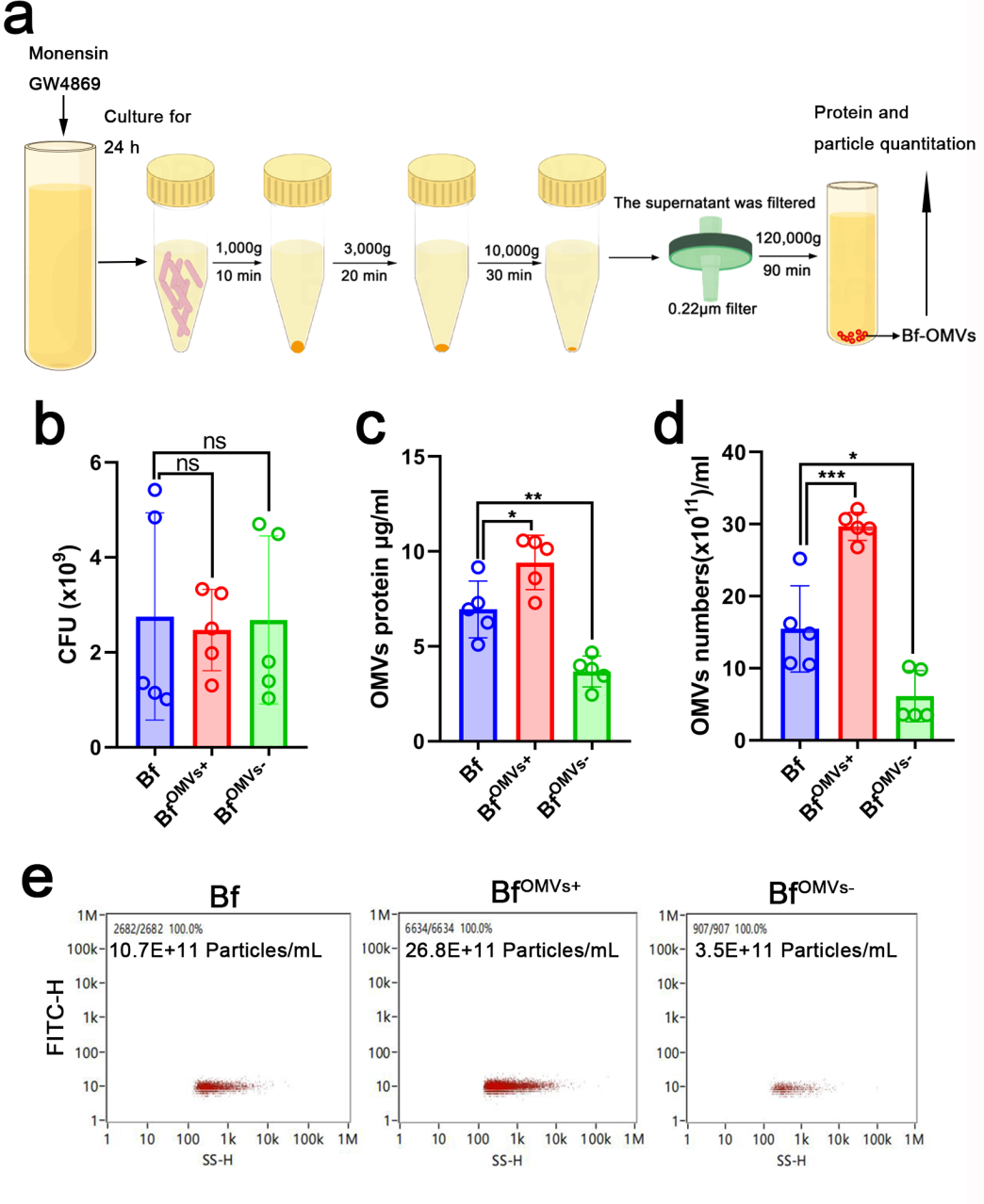


**Supplementary Figure S1. The bacteria were subjected to treatment with either monensin or GW4869 to stimulate or hinder the secretion of OMVs.** (a) Experiment design for testing the impact of monensin (Bf^OMVs+^) and GW4869 (Bf^OMVs-^) on the secretion of OMVs by *B. fragilis*. (b) Quantification of the number of bacterial colonies formed by the vehicle-, monensin-, or GW4869- treated *B. fragilis*. n = 5 per group. (c-e) Total protein contents and particle numbers of OMVs per milliliter of bacterial supernatant. n = 5 per group. **P* < 0.05, ***P* < 0.01, ****P* < 0.001, ns: no significance.


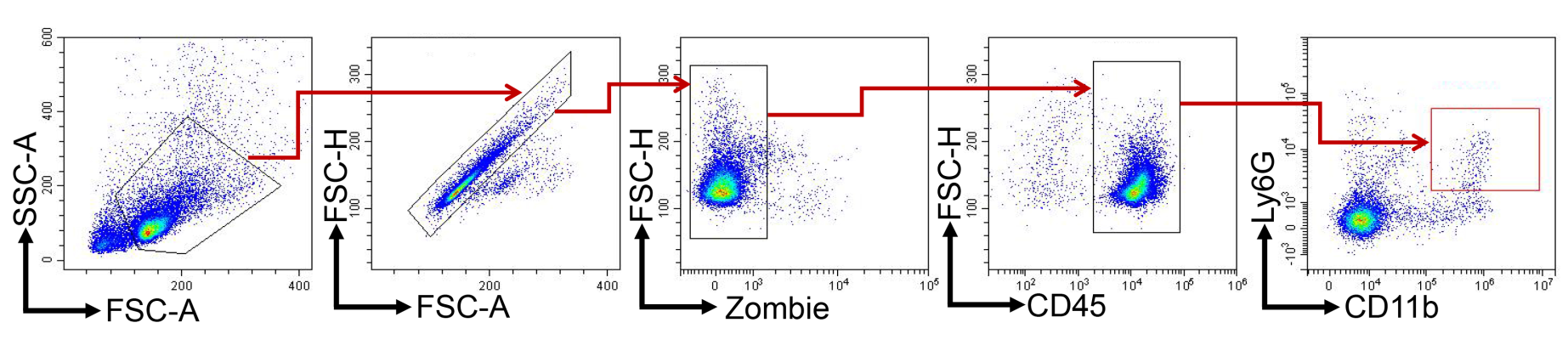


**Supplementary Figure S2. Flow cytometry gating strategy used for neutrophils. Neutrophils were identified as Zombie negative (live cells), CD45^+^CD11b^+^Ly6G^+^ cells.**


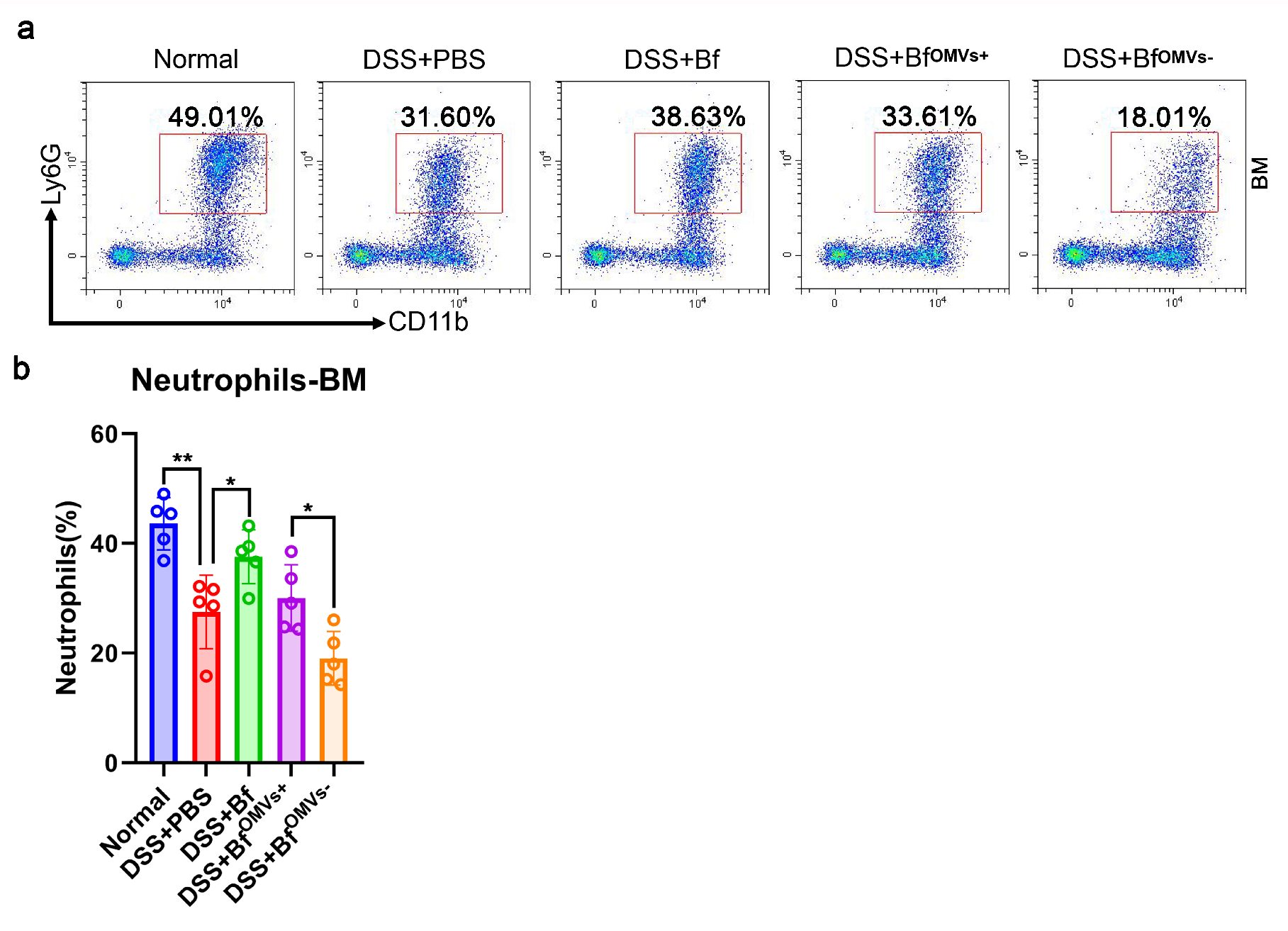


**Supplementary Fig****ure S3. Bf^OMVs+^ treatment alleviates peripheral neutrophil recruitment, restoring neutrophil levels in the bone marrows (BMs).** (a) Flow cytometric analysis of neutrophil percentages in the BMs. (b) Statistical analysis of neutrophil levels in the BMs. n = 5–8; results are presented as the mean ± SD; **P* < 0.05, ***P* < 0.01, *****P* < 0.0001.


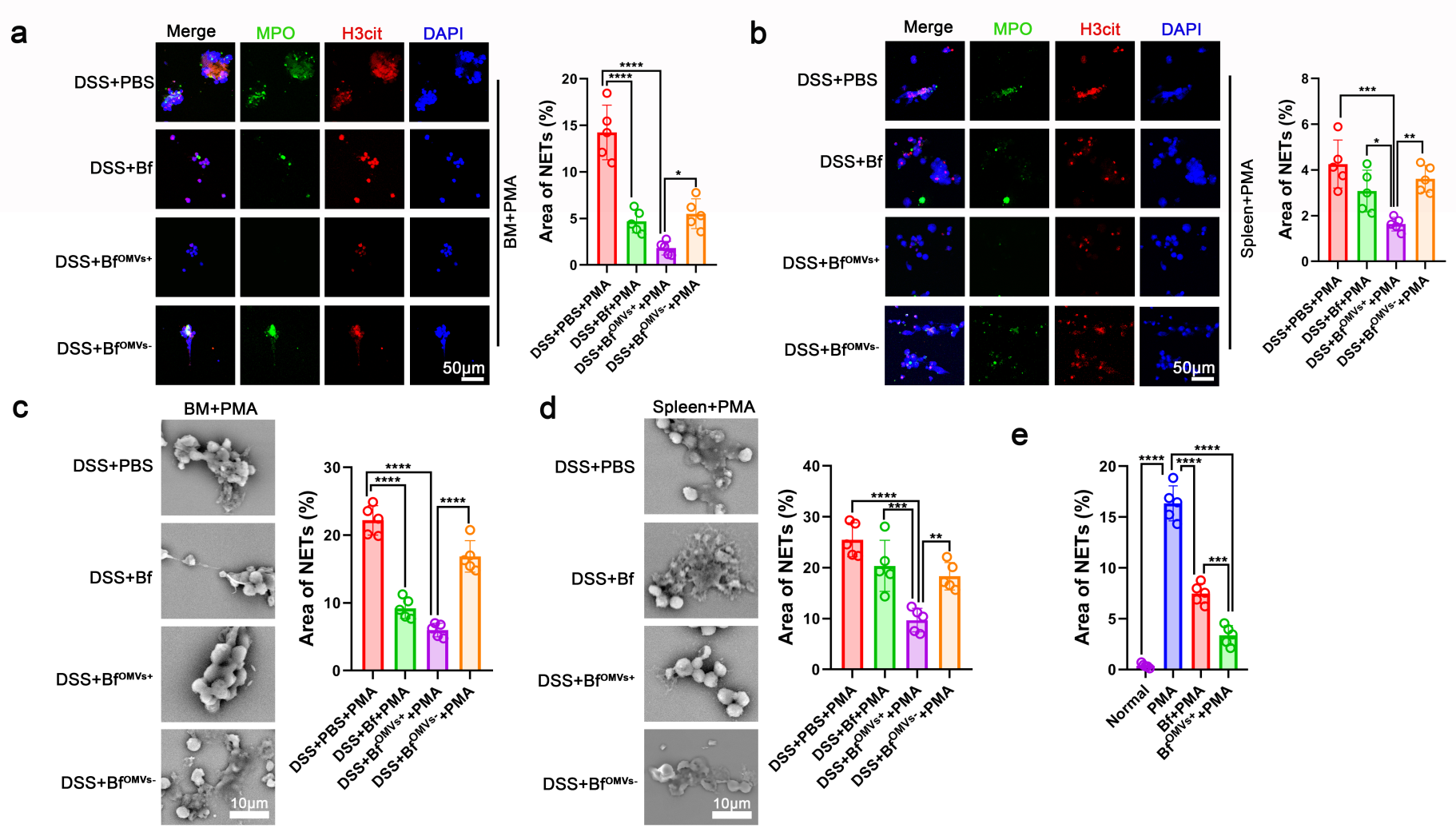


**Supplementary Figure S4. Bf^OMVs+^ inhibits PMA-induced NET formation.** (a, b) Neutrophils isolated from the BMs and spleens of colitis mice were cultured in vitro for 24 hours, with PMA added 4 hours before cell collection to induce NET formation. Immunofluorescence analysis was performed to evaluate NET formation. (c, d) SEM analysis was conducted to further assess NET formation after PMA treatment. (e) Neutrophils from the BM of normal mice were treated with *B. fragilis* and Bf^OMVs+^ while using PMA to induce NET formation in vitro. NETs were assessed by immunofluorescence, and the fluorescence area of NET expression was statistically quantified.  n = 5; results are presented as the mean ± SD; **P* < 0.05, ***P* < 0.01, ****P* < 0.001, *****P* < 0.0001.


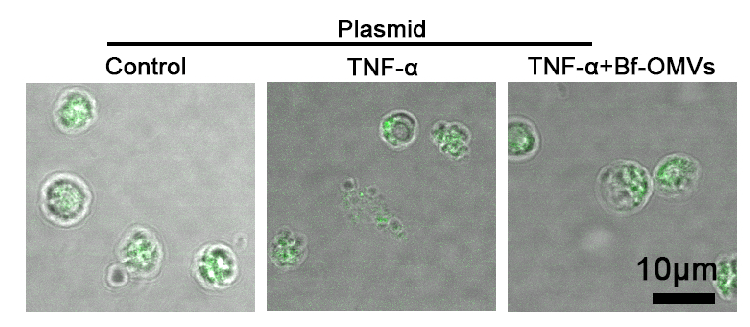


**Supplementary Figure S5. Bright field and fluorescence show mouse neutrophils transfected with plasmids expressing green fluorescent proteins, and positive cells (green fluorescence) were observed.**


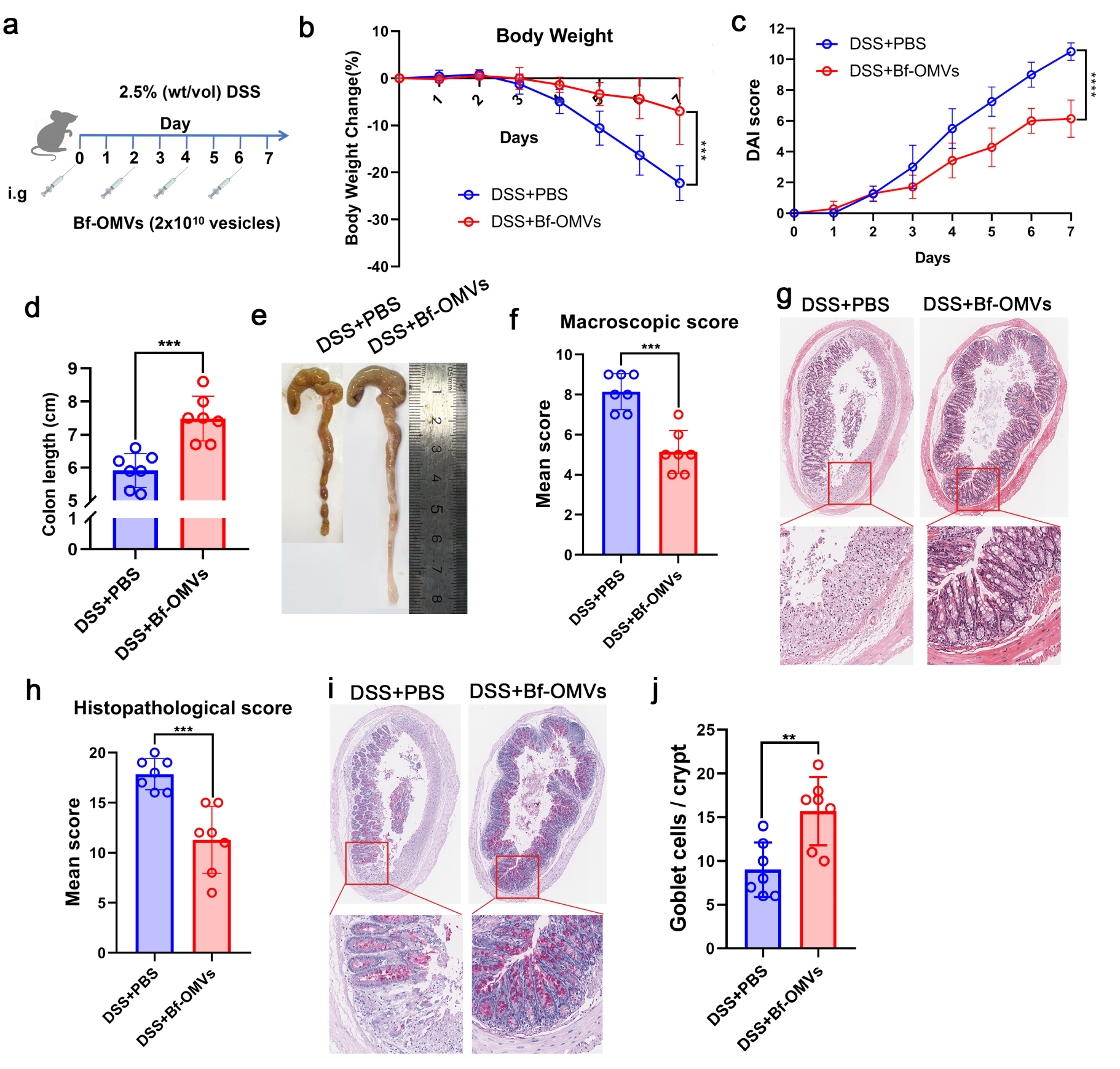


**Supplementary Figure S6. Bf-OMVs relieved DSS-induced colitis.** (a) Mice with DSS-induced colitis were treated with Bf-OMVs via oral gavage. (b) Body weight changes in the experimental groups, normalized to day 0 body weight. (c) DAI scores. (d) Colon length measurements. (e) Macroscopic appearance of the colon. (f) Macroscopic colon scores. (g) Histopathological examination of colon tissues stained with H&E. (h) Histopathological scores for the colon tissue samples were determined by H&E staining. (i, j) Alcian blue-periodic acid-Schiff (AB-PAS) staining used to assess goblet cell depletion, with goblet cell counts presented . n = 7; results are presented as the mean ± SD; ***P* < 0.01, ****P* < 0.001, *****P* < 0.0001.


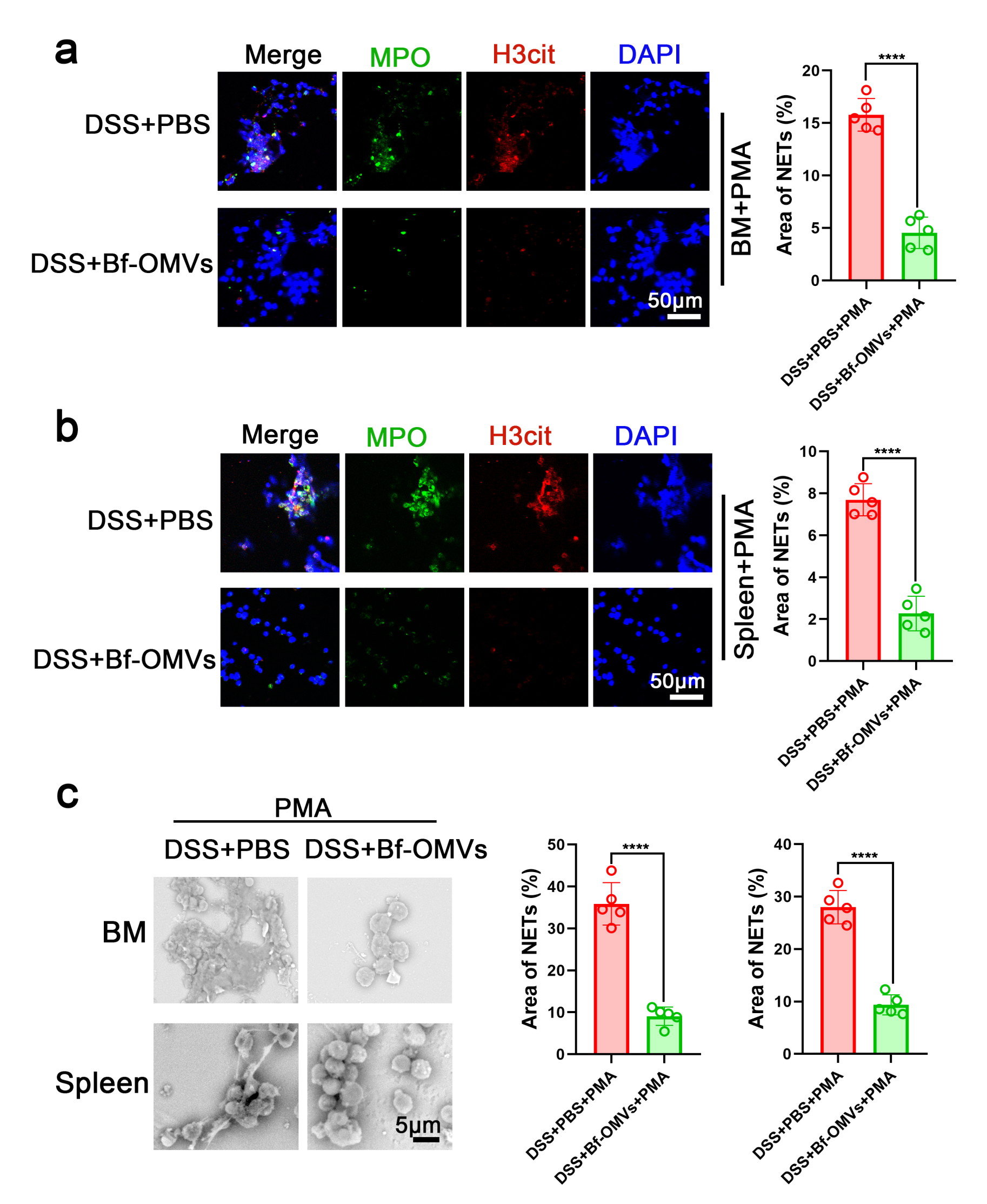


**Supplementary Figure S7. Bf-OMVs inhibit PMA-induced NET formation.** (a, b) Neutrophils isolated from the BMs and spleens of colitis mice were cultured in vitro for 24 hours, with PMA added 4 hours before cell collection to induce NET formation. Immunofluorescence analysis was performed to evaluate NET formation. (c) SEM analysis was conducted to further assess NET formation after PMA treatment. n = 5; results are presented as the mean ± SD; *****P* < 0.0001.


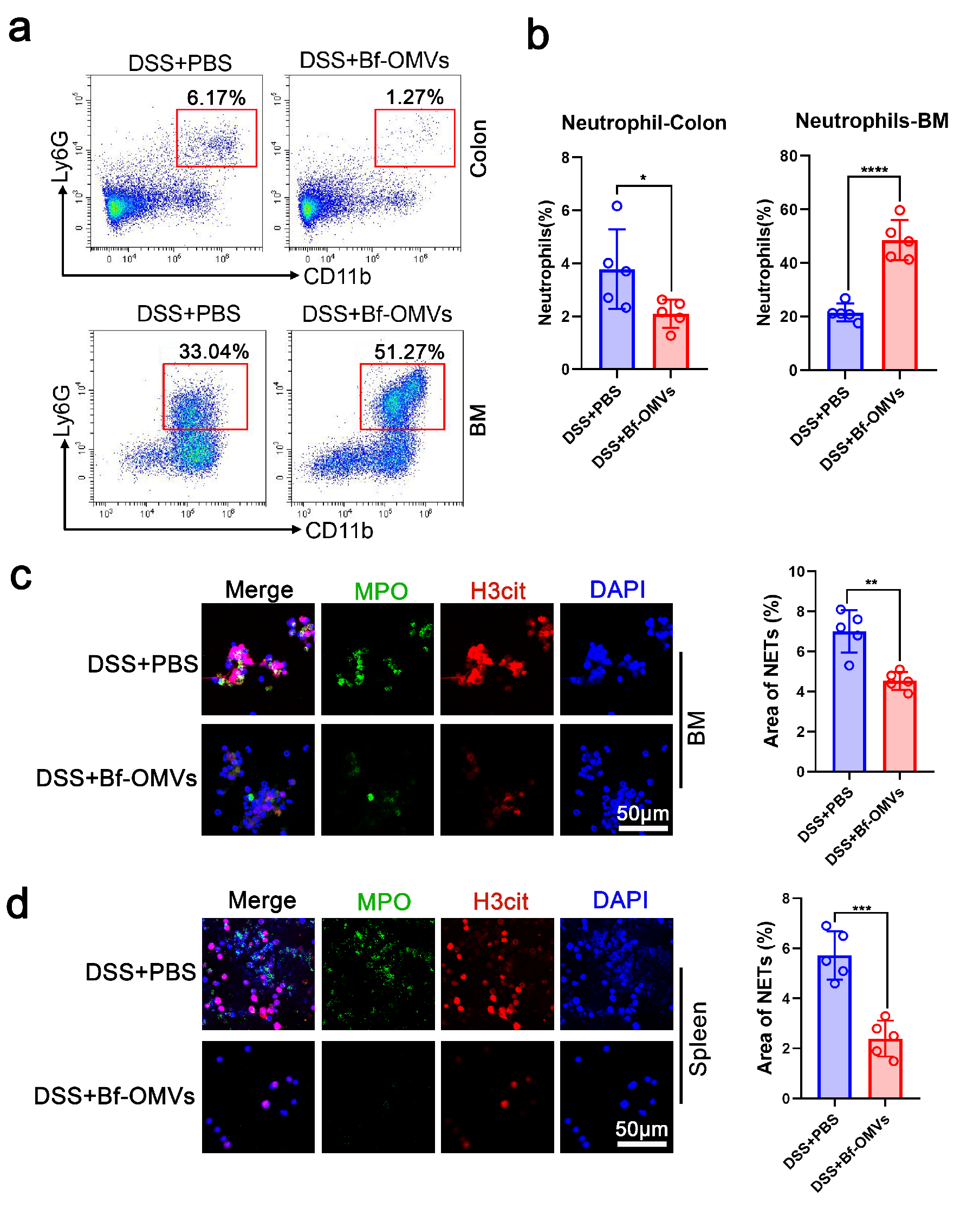


**Supplementary Figure S8. Bf-OMVs inhibit neutrophil recruitment and NET formation to alleviate DSS-induced colitis.** (a, b) Mice with DSS-induced colitis were treated with Bf-OMVs via oral gavage, the percentages of neutrophils in the colons and BMs were analyzed by flow cytometric analyses (a), and the results of the statistical analyses are shown (b). (c, d) Neutrophils isolated from the BMs and spleens of mice were cultured in vitro for 24 hours. Immunofluorescence analyse was used to assess NET formation. n = 5; results are presented as the mean ± SD; **P* < 0.05, ***P* < 0.01, ****P* < 0.001.


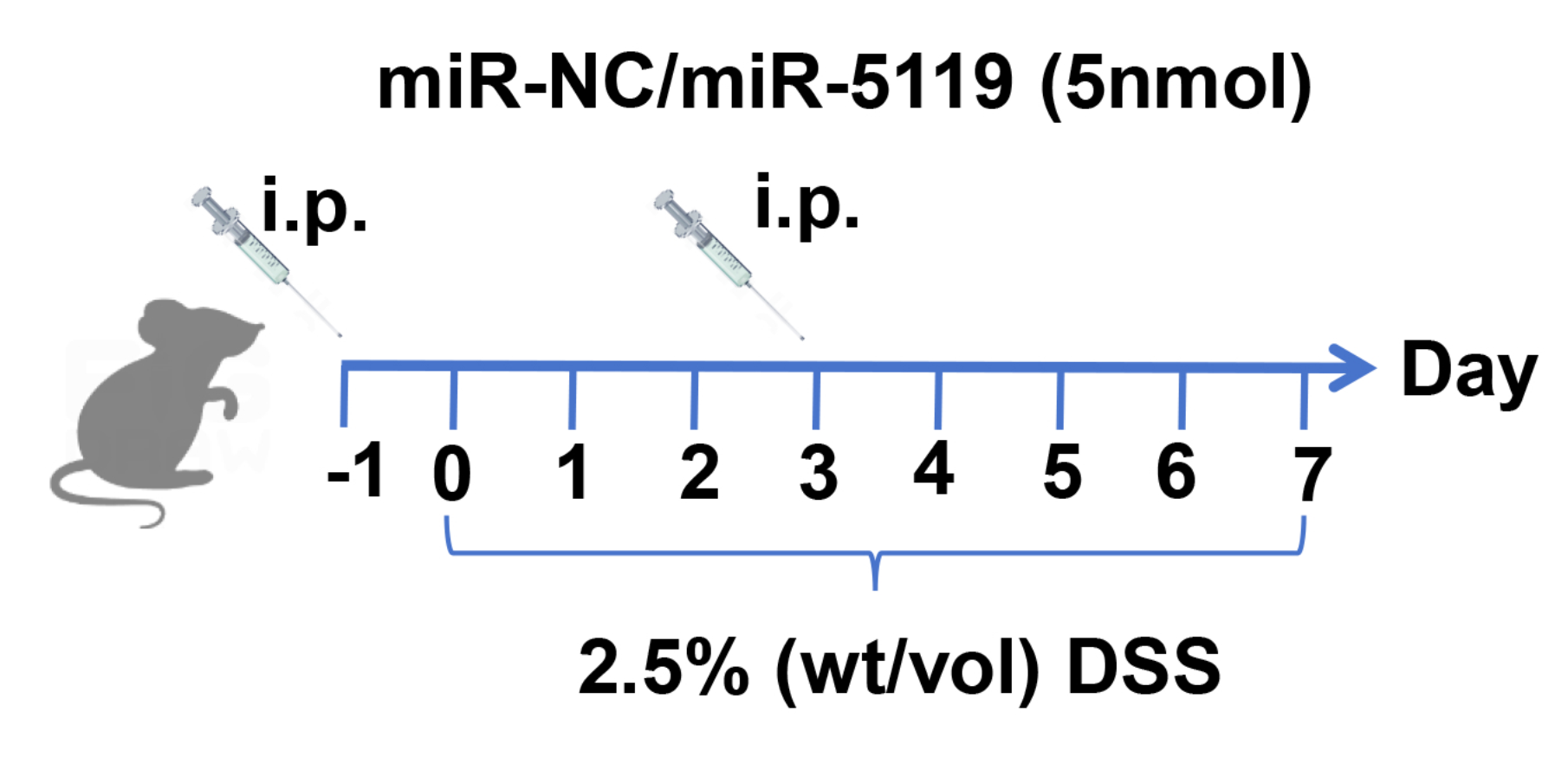


**Supplementary Figure S9. To further examine the therapeutic effect of miR-5119, we administered miR-5119 to mice with colitis via intraperitoneal injection.**


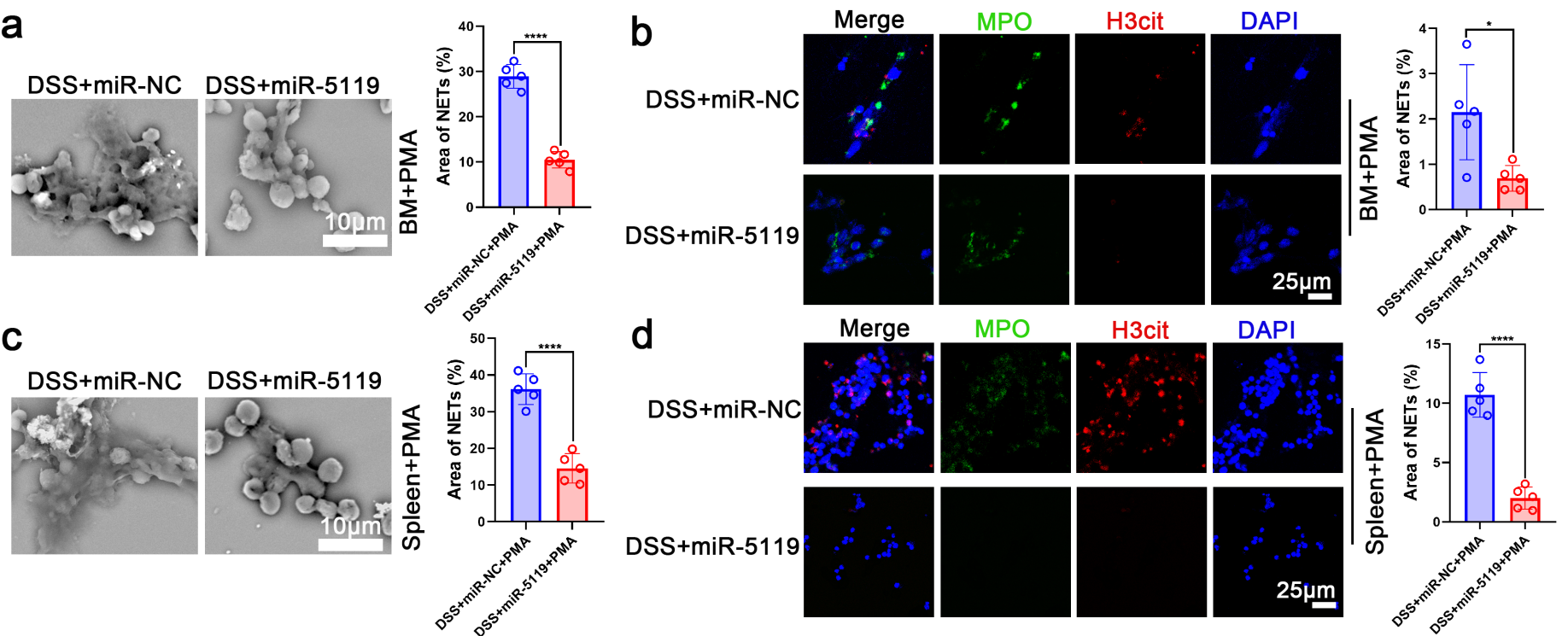


**Supplementary Figure S10. MiR-5119 delivery by Bf-OMVs inhibits NET formation in colitis mice.** (a, b) Neutrophils isolated from the BM of mice were cultured in vitro for 24 hours, and PMA was added 4 hours before cell collection to stimulate NET formation. SEM and immunofluorescence analysis were used to assess NET formation. (c, d) Neutrophils from the spleens of mice were similarly cultured and stimulated with PMA to evaluate NET formation using SEM and immunofluorescence. n = 5; results are presented as the mean ± SD; **P* < 0.05, *****P* < 0.0001.


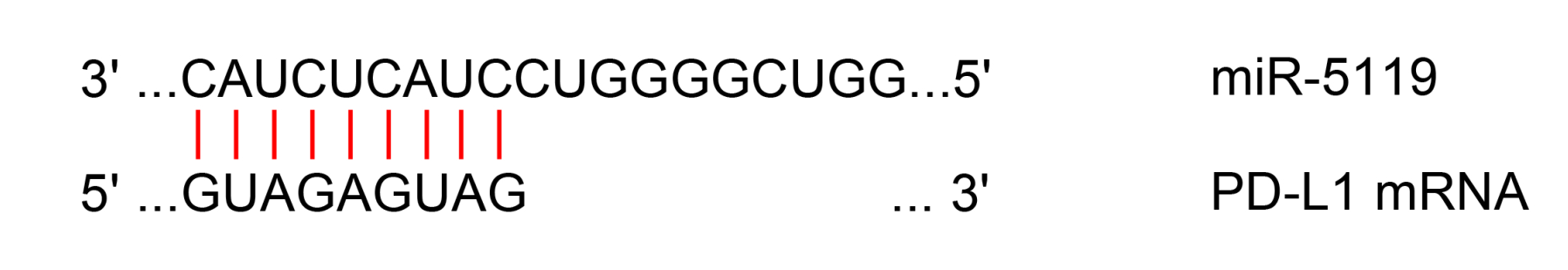


**Supplementary Figure S11. Predicted binding site of miR-5119 in the 3′-UTR of the PD-L1 gene.**


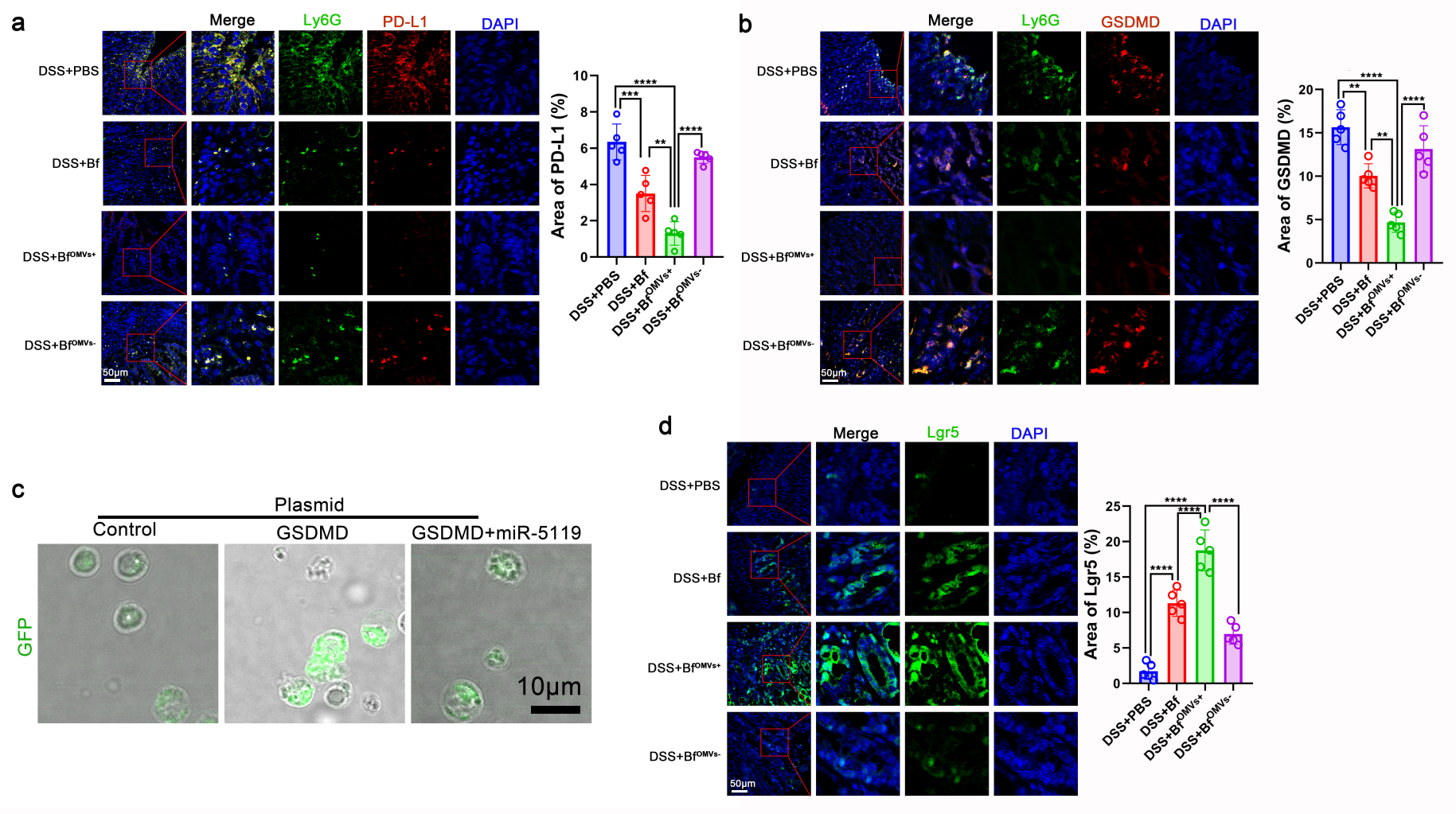


**Supplementary Figure S12. MiR-5119 inhibits NET formation by targeting PD-L1 to downregulate GSDMD-mediated NET release, thus alleviating DSS-induced colitis.**  (a) Immunofluorescence analyses were performed to detect PD-L1 expression in neutrophils in the colon, and the fluorescence area of PD-L1 expression was statistically quantified. (b) Immunofluorescence analyses were performed to detect GSDMD expression in neutrophils in the colon, and the fluorescence area of GSDMD expression was statistically quantified. (c) Bright field and fluorescence show mouse neutrophils transfected with plasmids expressing green fluorescent proteins, and positive cells (green fluorescence) were observed. (d) Lgr5^+^ cells in the intestinal mucosa were detected by immunofluorescence to assess intestinal stem cell (ISC) levels. n = 5; results are presented as the mean ± SD; ***P* < 0.01, ****P* < 0.001, *****P* < 0.0001.
